# Dynamic multimodal expressions support inference of both presence and relative salience of blended emotions

**DOI:** 10.1038/s41598-026-55904-z

**Published:** 2026-06-06

**Authors:** Alexandra Israelsson, Gustav Sandberg, Samuel Zeitler, Petri Laukka

**Affiliations:** 1 Department of Psychology, Stockholm University, 106 91 Stockholm, Sweden; 2 Department of Psychology, Uppsala University, 751 42 Uppsala, Sweden

**Keywords:** Blended emotions, Compound emotions, Facial expression, Mixed emotions, Multimodal expression, Non-linguistic vocalizations, Neuroscience, Psychology, Psychology

## Abstract

Few studies explore nonverbal communication of blended emotions, despite common reports of experiencing multiple emotions simultaneously. In two pre-registered experiments, we investigated if participants can accurately judge how prominently different emotions are expressed in dynamic multimodal portrayals of blended emotions. Actors portrayed pairwise combinations of anger, disgust, fear, happiness, and sadness in varying proportions, using facial gestures, body movement and vocal sounds. In Study 1, participants were instructed to choose two scales (out of 5 available scales: anger, disgust, fear, happiness, and sadness) that best described their impression of the emotional content of the portrayals, and rate how clearly both of the two chosen emotions were perceived. In Study 2, participants were instead free to choose any number of scales (out of the 5 available ones) in the emotion rating task. Results were consistent across both studies and showed that all blended emotions were accurately perceived with significantly higher ratings on scales corresponding to intended vs. non-intended emotions. Participants could also accurately judge the relative salience of each emotion, i.e., which was the dominant and the less dominant emotion in the blend. To summarize, results revealed nuanced perception of both presence and relative salience of blended emotions, which may help us navigate in a complex social environment.

**Supplementary Information:**

The online version contains supplementary material available at 10.1038/s41598-026-55904-z.

## Introduction

Perception research on nonverbal expressions has foremost focused on the recognition of single emotions, often by using pictures of facial expressions^1,2^. However, in real life, emotions are not static or only expressed through the face, but instead expressions evolve over time^3^ and are conveyed multimodally through the face, voice, and body^4,5^. Emotions may also not necessarily be expressed one at a time. Self-report studies on emotional experiences have repeatedly shown that individuals often feel more than one emotion simultaneously, which is called mixed, compound, or blended emotions^6–10^. Instances in which both positive and negative states occur simultaneously are commonly described as mixed emotions. These may be understood as a special case of blended or compound emotions, which is a broader concept referring to the simultaneous occurrence of multiple emotions irrespective of their emotional valence^11–13^. This raises the question if individuals may not only experience, but also express and perceive, multiple emotions at the same time—which is the topic of this paper. More specifically, we extend previous work^14^ by investigating if individuals are able to perceive how prominently different emotions are expressed in dynamic multimodal portrayals of blended emotions.

Although research on nonverbal expression is currently expanding the number and complexity of emotions under study^15,16^, studies on perception of blended emotions remain limited. One line of research has indirectly studied blended emotions by rating facial expressions of single emotions on multiple emotion scales. These studies showed that individuals sometimes perceive more than one emotion from single emotion expressions and may also agree upon the other, non-intended emotions^17–20^. One explanation for these findings is that different emotions may be associated with similar facial features^21^, but it could also be the case that some facial stimuli contain features from several emotions although they were intended as expressions of a single emotion.

Initial work that directly investigated perception of facial expressions of blended emotions was performed by Nummenmaa^22,23^. He asked actors to express pairwise combinations of emotions, and showed that many emotion pairs were indeed recognizable from photographs. In the first study, accurate recognition was reported for all pairwise combinations of anger, pleasure, and surprise^22^, and for 6 out of 10 combinations of fear, hate, pleasure, sorrow, and surprise in a follow-up study^23^. These findings are intriguing, but also limited by the use of only one actor per study and the reliance on forced-choice tasks, which might not fully capture the complexity of blended emotion perception. More recently, Du et al.^24^ created a more comprehensive database of photographs of blended facial expressions, wherein 230 participants expressed 15 different emotion combinations (e.g., angrily surprised). It should be noted that the participants were not free to express the blended emotions in any way that they seemed fit, but were rather instructed by the authors to combine certain facial features associated with different emotions. Du et al.^24^ conducted machine learning analyses which confirmed that the facial features differed systematically between the different blended emotions. Similar results were obtained in Guo et al.^25^, who evaluated another database consisting of photographs of pairwise combinations of 7 emotions using various machine learning methods. A human validation of the stimuli from Du et al.^24^ was reported by Pallett and Martinez^26^, who showed that approximately half (7 out of 15) of the combinations were accurately labelled.

Rather than to use human stimuli, another approach is to study perception of blended emotions by creating synthetic facial emotion expressions using various methods^27–29^. For example, Mäkäräinen et al.^30^ used a physically based facial model to combine various features associated with single emotions. They created synthetic faces displaying combinations of two emotions that changed dynamically from neutral to peak expression. In perception studies, their results showed that only one pairwise combination (happiness–surprise) was accurately recognized, whereas cross-valence combinations were primarily perceived as other complex emotions, and same-valence combinations as single emotions.

Turning next to vocally expressed emotions, we note that studies on blended emotions are rare. Nevertheless, Juslin et al.^31^ found that in naturalistic vocal recordings, the speakers themselves frequently reported that they had experienced blended emotions during the recording. However, when the recordings were presented to listeners, they generally perceived these as single emotions (although a few emotion combinations were indeed recognized with above chance accuracy). Synthesis methods have also been employed in vocal research. Burkhardt and Weiss^32^ manipulated two different sets of acoustic features to synthesize pairwise combinations of emotions. In perception studies, human judges were generally able to recognize one, but not the other, of the two intended emotions. Accurate recognition corresponded with the emotions synthesized with one of the feature sets, which seemingly contained more efficient features (pitch and duration) than the other set (voice quality and articulation). Recently, Zhou et al.^33^ used data driven speech synthesis methods to create blended vocal expressions, and reported that human judges were able to perceive anger, happiness, and sadness when these emotions were combined with expressions of surprise to create pairwise combinations.

Research on bodily expression of emotion is less extensive than research on facial and vocal expressions. Studies suggest that both movements and postures may convey emotion-specific cues that can be reliably recognized even in the absence of other expressive channels (e.g.,^34–36^, for reviews, see^37,38^). However, the role of bodily expression in conveying blended emotions has received little empirical attention.

In recent study by Israelsson et al.^14^, actors were instructed to express blended emotions consisting of all pairwise combinations of anger, disgust, fear, happiness, and sadness—using facial gestures, body movement, and vocal sounds—with the intention that both emotions should be equally prominent in the resulting expression. Results from perception studies using a combined forced-choice and rating scale task first showed that all blended emotions could be accurately recognized from the dynamic multimodal expressions. In a second experiment, the stimuli were instead presented in visual-only and auditory-only conditions, and all blended emotions were again accurately perceived, although accuracy was lower in the auditory-only condition.

In summary, previous studies suggest that at least some blended emotions can be expressed and perceived from both facial and vocal nonverbal emotion expressions, although some mixed results also occur. The current study is intended as a follow-up to Israelsson et al.^14^, which like other previous efforts contended with investigating if both emotions can be accurately perceived from pairwise emotion combinations. In the current study, we expand upon previous research by offering an initial investigation into whether the relative salience of blended emotions can also be perceived. Depending on the emotion eliciting situation, an individual who experiences, for example, both anger and sadness at the same time, may still feel more angry than sad or vice versa. Establishing whether the relative salience of blended emotions can be expressed and perceived therefore presents a key next step in advancing research on nonverbal emotion communication. Accordingly, we performed two pre-registered studies to investigate if it is possible to accurately judge both the presence (i.e., which emotions are included in the blend) and the relative salience (i.e., how much of each emotion is included in the blend) using dynamic and multimodal emotion expressions. Whereas Study 1 examined whether observers could infer the presence of two emotions under constrained response conditions, Study 2 examined whether similar judgments would emerge under less constrained rating conditions, allowing us to examine the robustness of blended emotion perception across different response formats.

## Study 1

In Study 1, we investigate if participants can accurately judge how prominently different emotions are expressed in dynamic multimodal portrayals of pairwise emotion combinations with varying proportions. All analyses were pre-registered (https://osf.io/ngazb) except when noted otherwise, and we report all manipulations, measures, and participants exclusions. Data and code are available on the Open Science Framework (https://osf.io/pws8u).

### Method

#### Participants

Forty-three adults (27 women) who were between 20 and 45 years (*M* = 26.6, *SD* = 6.4) took part in the experiment. The sample size was determined apriori using G*Power^39^, and was based on power analysis for one-way repeated measures analysis of variance (ANOVA) with 10 measurements. The goal was to obtain 0.95 power to detect a medium effect size (f = 0.25) at standard 0.05 alpha error probability. The correlation between repeated measures was conservatively set to 0 and the nonsphericity correction epsilon was set to 1. These parameters were chosen to provide a lower-bound estimate of the study’s power. Using these settings, G*Power suggested a sample size of N = 39. No participants were excluded after data collection.

Participants were recruited through advertisements on online recruitment sites and were compensated with two movie tickets. Informed consent was collected online from each participant. Only individuals between 18 and 65 years and who understood Swedish were eligible for participation. All methods were performed in accordance with the relevant guidelines and regulations, and in accordance with the Declaration of Helsinki. The study was approved by the Swedish Ethical Review Authority (decision no. 2021-00972).

#### Emotion portrayals

We used portrayals of blended emotions which were recorded as part of a larger project on dynamic multimodal emotion expression. Actors were instructed to express all pairwise combinations of anger, disgust, fear, happiness, and sadness (i.e., 10 emotion combinations) using facial gestures, body movements and vocal sounds (see^14^, for detailed information about recording procedures). Combinations of these emotions occur in self-reports of subjective feelings^7^, and they are also well recognized as single emotions^40,41^.

More specifically, the actors were instructed that the emotion combinations should be expressed in varying proportions: 30:70, 50:50 and 70:30. For expressions in the 30:70 condition, the first emotion should be less prominent than the second (e.g., for “30:70 anger–happiness”, anger should be less prominent than happiness). For 50:50 expressions, both emotions should instead be equally prominent in the resulting expression (e.g., “50:50 fear–sadness”). Finally, in the 70:30 condition, the first emotion should be more prominent than the second (e.g., for “70:30 disgust–happiness”, disgust should be more prominent than happiness). It should be noted that although the conditions with different proportions are named 30:70 and 70:30, this does not mean that we intended for the actors to express exactly 30% of one emotion and 70% of the other. Rather this terminology simply means that the emotion with the smaller proportion should be less prominent than the emotion with the larger proportion in the resulting blended expression.

All actors were provided with the same instructions, specifying that the emotion combinations should be conveyed simultaneously through all channels (face/body and voice). They were also instructed that the expressions should be as convincing as possible, but without using overtly stereotypical expressions. Regarding vocal sounds, the actors were free to choose any non-linguistic vocalization (e.g., cries, laughter, groans; see Scherer^42^), but no real or fake words were allowed. The instructions further included definitions and scenarios for each single emotion based on previous studies^43^. The actors’ intended emotion combinations were used as ground truth for determining whether the participants’ emotion judgments were correct or incorrect.

Recordings were conducted in a room with studio lighting and dampened acoustics, using a setup with a high-quality camera (Blackmagic Pocket Cinema Camera 6 K, Blackmagic Design Pty Ltd, Port Melbourne, Australia), lens (Sigma 35 mm F1.4 DG HSM, Sigma Corporation, Kawasaki, Japan) and microphone (Røde NTG3B, Røde Microphones, Sydney, Australia). The audio level was calibrated relative to the loudest expected level and then kept constant during the recording session. For more details of the recording procedure, see Israelsson et al.^14^.

**Fig. 1:**
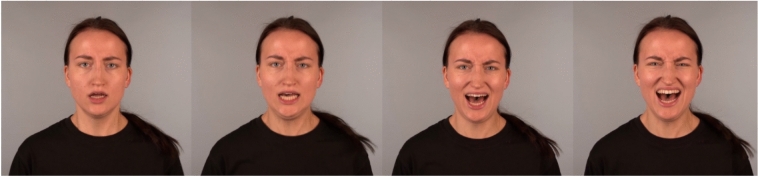
Examples of stills from the video recordings showing an actor portraying a combination of anger and fear where both emotions appear with equal proportions. *Note.* From “Blended Emotions can be Accurately Perceived from Dynamic Facial and Vocal Expressions,” by A. Israelsson, A. Seiger and P. Laukka, 2023, *Journal of Nonverbal Behavior, 47*(3), p. 271 (https://doi.org/10.1007/s10919-023-00426-9). CC BY 4.0.

We selected recordings from 6 actors (3 women) and each actor provided 30 recordings (i.e., 30:70, 50:50, and 70:30 expressions of each of the 10 different emotion combinations), for a total of 180 brief (1–5 s) video stimuli. The actors were selected based on the recognition accuracy of their 50:50 expressions in a previous study^14^, but accuracy for the 30:70 and 70:30 expressions had not been assessed previously. Each video showed a frontal view of the actor’s upper-torso and face. Figure 1 shows examples of stills from the videos.

#### Emotion perception task

All participants rated all of the 180 video clips described above in an online experiment. The experiment was constructed in PsychoPy^44^ and was presented online using the Pavlovia (https://pavlovia.org/) platform. Stimulus duration ranged between 1 and 5 s and stimulus size was normalized in PsychoPy using ‘norm’ units with a size of 1 × 1, corresponding to approximately 50% of the screen width and height, ensuring that video size was proportional across different screen resolutions. The task could only be completed on a computer and was not compatible with mobile phones or tablets.

Following Israelsson et al.^14^, the emotion perception task combined elements of forced-choice and rating scale tasks in order to assess both which emotions were perceived and how clearly they were perceived. For each video, participants were shown 5 rating scales (anger, disgust, fear, happiness, and sadness). They then had to choose 2 out of the 5 available scales, based on their impression of the emotional content of the video, and use these scales to indicate how clearly each of the selected two emotions were perceived. The rating scales ranged from 0 (emotion not at all prominent) to 10 (emotion very prominent). Stimuli were presented in random order (different for each participant) to avoid order effects.

The task included step-by-step instructions and participants were also provided the same definitions of the included emotions as were given to the actors. Participants were instructed to use headphones and to adjust the sound level to a comfortably loud level during three practice trials presented before the experiment began. No formal headphone screening, automated sound-on verification, or attention-checks were included in the task. The task was self-paced and participants were allowed to replay the videos as many times as needed to perform the ratings. Response time and number of views were not recorded. The whole experiment took around 45–60 min to complete.

### Results

#### Emotion ratings

Table 1 presents the emotion ratings for all pairwise emotion combinations and for all rating scales. The ratings were first analyzed using one-way repeated measures ANOVAs, with the intended emotion combination (10 levels: anger–disgust, anger–fear, anger–happiness, anger–sadness, disgust–fear, disgust–happiness, disgust–sadness, fear–happiness, fear–sadness, happiness–sadness) as a within-subjects variable. The ANOVAs were performed separately for the participants’ mean ratings on each rating scale (anger, fear, disgust, happiness, and sadness) and for each stimulus proportion (30:70, 50:50 and 70:30). Significant main effects (*p*s < .001) showed that the ratings varied systematically as a function of emotion combination for all rating scales in all three stimulus proportion conditions (see Table 2).

**Table 1:** Emotion ratings for each emotion combination and emotion proportion (Study 1).

Emotion combination	Emotion ratings (M/SD)				
	Anger	Disgust	Fear	Happiness	Sadness
Ang–disg					
30:70*	**3.67 (0.96)**	**6.95 (1.32)**	0.74 (0.87)	0.31 (0.65)	0.69 (0.70)
50:50	**4.94 (1.27)**	**5.33 (1.33)**	0.87 (1.08)	0.17 (0.61)	0.46 (0.72)
70:30*	**6.24 (1.06)**	**3.69 (1.28)**	0.62 (0.64)	0.17 (0.65)	0.53 (0.68)
Ang–fea					
30:70	**4.97 (1.08)**	0.86 (0.87)	**3.93 (1.05)**	0.10 (0.68)	2.05 (0.89)
50:50	**7.99 (1.33)**	0.80 (0.69)	**2.34 (1.11)**	0.15 (0.62)	1.10 (0.93)
70:30*	**8.47 (0.88)**	1.10 (0.91)	**2.12 (1.24)**	0.07 (0.64)	0.43 (0.77)
Ang–hap					
30:70*	**2.98 (0.92)**	0.24 (0.71)	0.57 (0.63)	**7.14 (1.38)**	0.24 (0.63)
50:50	**5.07 (1.64)**	0.46 (0.82)	0.26 (0.71)	**5.80 (1.69)**	0.13 (0.73)
70:30*	**6.10 (1.09)**	0.77 (1.19)	0.91 (0.79)	**2.91 (1.37)**	0.40 (0.61)
Ang–sad					
30:70*	**5.16 (1.57)**	0.29 (0.73)	0.74 (0.91)	0.23 (0.68)	**6.88 (1.30)**
50:50	**6.88 (1.08)**	0.53 (0.68)	1.23 (1.02)	0.12 (0.56)	**4.24 (1.18)**
70:30*	**6.55 (0.82)**	0.91 (0.89)	1.06 (0.78)	0.50 (0.57)	**2.79 (1.12)**
Disg–fea					
30:70*	0.76 (0.64)	**4.40 (1.39)**	**6.03 (1.12)**	0.16 (0.65)	0.71 (0.94)
50:50	1.73 (0.91)	**6.43 (1.26)**	**2.83 (1.01)**	0.32 (0.58)	1.35 (1.06)
70:30*	2.26 (1.02)	**7.38 (1.42)**	**1.64 (1.16)**	0.14 (0.67)	1.36 (1.13)
Disg–hap					
30:70*	0.29 (0.80)	**2.25 (1.36)**	0.78 (0.83)	**7.55 (1.38)**	0.85 (0.75)
50:50	0.35 (0.79)	**4.40 (1.60)**	0.61 (0.85)	**5.96 (1.72)**	0.86 (1.09)
70:30*	0.93 (1.14)	**6.20 (1.55)**	1.70 (1.10)	**2.36 (1.29)**	0.88 (1.02)
Disg–sad					
30:70*	0.89 (0.60)	**1.71 (0.95)**	1.48 (0.87)	0.07 (0.60)	**7.21 (1.19)**
50:50	0.91 (0.75)	**4.44 (1.26)**	1.95 (0.97)	0.11 (0.68)	**4.76 (1.24)**
70:30*	0.89 (0.75)	**4.38 (1.00)**	2.33 (0.74)	0.10 (0.78)	**3.48 (1.03)**
Fea–hap					
30:70*	0.61 (0.93)	0.20 (0.58)	**2.78 (1.21)**	**7.14 (1.56)**	0.48 (0.67)
50:50	0.34 (0.69)	0.53 (0.78)	**4.33 (1.16)**	**5.62 (1.38)**	0.48 (0.78)
70:30*	0.38 (0.60)	1.45 (0.97)	**4.99 (1.50)**	**2.71 (0.99)**	1.28 (1.05)
Fea–sad					
30:70*	0.60 (0.74)	0.33 (0.57)	**3.34 (1.07)**	0.06 (0.75)	**7.77 (1.05)**
50:50	0.53 (0.77)	0.69 (0.56)	**5.36 (1.47)**	0.05 (0.67)	**5.95 (1.18)**
70:30*	0.44 (0.72)	0.85 (0.86)	**6.04 (1.26)**	0.20 (0.70)	**3.91 (1.39)**
Hap–sad					
30:70*	0.35 (0.66)	0.29 (0.70)	1.65 (0.81)	**2.23 (0.88)**	**6.59 (1.26)**
50:50	0.46 (0.64)	0.28 (0.68)	0.49 (0.72)	**5.21 (1.35)**	**4.74 (1.32)**
70:30	0.31 (0.72)	0.09 (0.66)	0.79 (0.80)	**5.60 (1.37)**	**4.62 (1.60)**

*N* = 43. ang = anger, disg = disgust, fea = fear, hap = happiness, sad = sadness. 30:70 = The first emotion is intended to be less prominent than the second emotion. 50:50 = Both emotions are intended to be equally prominent. 70:30 = The first emotion is intended to be more prominent than the second emotion. Values in bold indicate emotion combinations that contain emotions that correspond to the rating scale (i.e., target combinations). Asterisks indicate emotion combinations where proportions between emotions were accurately judged (i.e., significantly higher ratings for the more vs. the less prominent emotion) as indicated by post-hoc repeated measures *t*-tests, *p*s < .0025). Emotion ratings: 0 = Emotion not at all prominent, 10 = Emotion very prominent.

**Table 2:** F-statistics and effect size estimates for all emotion scales and proportion combinations (Study 1).

Emotion scale [emotion proportion]	*F*(9, 378)	*p*	η^2^_*p*_	η^2^_*G*_
Anger				
[30:70]	191.20	< .001	0.82	0.75
[50:50]	344.58	< .001	0.89	0.84
[70:30]	533.08	< .001	0.93	0.88
Disgust				
[30:70]	231.02	< .001	0.85	0.78
[50:50]	243.52	< .001	0.85	0.79
[70:30]	231.72	< .001	0.85	0.77
Fear				
[30:70]	152.06	< .001	0.78	0.71
[50:50]	114.81	< .001	0.73	0.66
[70:30]	131.98	< .001	0.76	0.69
Happiness				
[30:70]	482.78	< .001	0.92	0.89
[50:50]	281.53	< .001	0.87	0.81
[70:30]	160.04	< .001	0.79	0.72
Sadness				
[30:70]	498.70	< .001	0.92	0.88
[50:50]	189.62	< .001	0.82	0.76
[70:30]	93.58	< .001	0.69	0.59

*N* = 43. Mauchly’s test of sphericity showed that none of the emotion scales met the assumption of sphericity, but effects remained statistically significantly after Greenhouse–Geisser correction (all corrected *ps* < 0.0001). We used generalized eta squared (η^2^_*G*_) to estimate the variation explained by the within-subjects factor while accounting for both number of factors and individual differences (which enables comparison across studies regardless of design), and partial eta squared (η^2^_*p*_) to estimate the amount of variation explained by the within-subjects factor when controlling for the number of factors.

An inspection of Table 1 reveals that target emotion combinations (i.e., where the rating scale matches with one of the intended emotions) in general received higher ratings than non-target emotion combinations (i.e., where the scale and intended emotions do not match). For example, in the column for anger ratings, one can see that the target combination 30:70 anger–disgust (M = 3.67) received higher ratings than the non-target combination 30:70 disgust–fear (M = 0.76). With a couple of exceptions, post-hoc multiple comparisons (repeated measures *t*-tests) showed that the differences between target and non-target emotion combinations were significant for each rating scale within each stimulus proportion (*p*s < .0001, Bonferroni corrected alpha level = 0.0021, i.e., corrected for 24 comparisons per ANOVA). The exceptions were observed for ratings of fear, where the target 50:50 anger–fear (M = 2.34) was not rated significantly higher than the non-target 50:50 disgust–sadness (M = 1.95), and the targets 70:30 anger–fear (M = 2.12) and 70:30 disgust–fear (M = 1.64) were rated lower (instead of higher) than the non-target 70:30 disgust–sadness (M = 2.33). Details about the target versus non-target comparisons are presented in Table S1.

##### Exploratory analyses

We further investigated if individuals were able to correctly judge the proportions in the 30:70 and 70:30 conditions, which means that the more prominent emotion in each pairwise combination should receive higher ratings than the less prominent emotion. As seen in Table 1, this was the case for most of the blends in the 30:70 and 70:30 combinations (50:50 combinations were not included in these analyses), as indicated by post-hoc multiple comparisons (repeated measures *t*-tests with alpha = 0.0025, i.e., corrected for 20 comparisons). For example, Table 1 shows that for 30:70 anger–disgust, disgust ratings (M = 6.95) ratings were significantly higher than the anger ratings (M = 3.67). Exceptions to this trend were observed for the 70:30 happiness–sadness combination where happiness ratings (M = 5.60) were not significantly higher than the sadness ratings (M = 4.62, *p* = .009) and for the 30:70 anger–fear combination where anger was actually rated higher (M = 4.97) than fear (M = 3.93, *p* < .001). Overall, this suggests that individuals were mostly able to accurately perceive the intended proportions of the emotions in the 30:70 and 70:30 conditions (the results from the exploratory analyses are presented in full in Table S2).

#### Accuracy indices

Emotion ratings were further used to calculate three indices of emotion recognition accuracy^31^. *Generous accuracy* is a measure of the ability to correctly perceive at least one of the two intended emotions, and *strict accuracy* is a measure of the ability to correctly perceive both of the two intended emotions. *Very strict accuracy* is introduced in the current study and is a measure of the ability to correctly perceive both of the two intended emotions as well as their proportions (this index only applies to the 30:70 and 70:30 conditions). For generous accuracy, a response was scored as correct if either of the used rating scales corresponded to either of the two intended emotions of a stimulus. For strict accuracy, a response was only scored as correct if the chosen rating scales corresponded to both of the two intended emotions of a stimulus. For very strict accuracy, finally, a response was only scored as correct if the chosen rating scales corresponded to both of the two intended emotions, and if the more prominent of the two intended emotions received a higher rating than the less prominent intended emotion.

For all three indices, correct responses were coded as 1 and incorrect responses as 0, and recognition accuracy was then calculated as the proportion of correct responses. For generous accuracy, the probability of obtaining no correct responses was calculated as the probability of selecting two incorrect alternatives out of the three incorrect response options, yielding 3 possible incorrect response pairs out of 10 total possible response pairs (3/10 = 0.30). Therefore, the probability of obtaining at least one correct response by chance was 1–0.30 = 0.70. For strict accuracy, the probability of getting the first emotion correct is 2/5, and the probability of getting the second emotion right is 1/(5–1) = 0.25. The combined probability of getting both emotions right by chance is thus 0.10. For very strict accuracy, finally, the probability of getting both of the intended emotions, and their proportions, correct by chance is (2/5)*(1/4)*(1/2) = 0.05.

Averaged across all emotions, both strict accuracy (M = 0.55, SD = 0.50, 95% CI [0.53, 0.56]) and very strict accuracy (M = 0.32, SD = 0.47, 95% CI [0.31, 0.33]) were significantly above chance, as indicated by 95% CIs not overlapping with the chance level of each index. Table 3 presents accuracy values for each condition and shows that all three indices were significantly higher than chance for all emotion combinations (with one exception), again indicated by 95% CIs that did not overlap with chance. The one exception was observed for very strict accuracy for 70:30 disgust–sadness, which was not significantly higher than chance (M = 0.09, 95% CI [0.053, 0.12]).

**Table 3:** Emotion recognition accuracy for each emotion combination and emotion proportion (Study 1).

Emotion combination	Emotion recognition accuracy					
	Very strict^a^	95% CI	Strict	95% CI	Generous	95% CI
Ang–disg						
30:70	0.38	[0.33, 0.43]	0.59	[0.54, 0.64]	0.99	[0.98, 1.00]
50:50	NA	NA	0.64	[0.57, 0.71]	1.00	[0.99, 1.00]
70:30	0.36	[0.29, 0.42]	0.62	[0.56, 0.69]	0.99	[0.98, 1.00]
Ang–fea						
30:70	0.17	[0.12, 0.22]	0.42	[0.37, 0.48]	0.96	[0.93, 0.98]
50:50	NA	NA	0.47	[0.41, 0.53]	0.99	[0.98, 1.01]
70:30	0.39	[0.31, 0.46]	0.47	[0.39, 0.55]	1.00	[1.00, 1.00]
Ang–hap						
30:70	0.52	[0.44, 0.59]	0.69	[0.62, 0.76]	0.99	[0.98, 1.00]
50:50	NA	NA	0.75	[0.67, 0.83]	1.00	[0.99, 1.00]
70:30	0.36	[0.27, 0.44]	0.53	[0.43, 0.62]	0.98	[0.96, 1.00]
Ang–sad						
30:70	0.33	[0.27, 0.38]	0.70	[0.64, 0.75]	1.00	[1.00, 1.00]
50:50	NA	NA	0.62	[0.56, 0.69]	0.98	[0.96, 1.00]
70:30	0.25	[0.20, 0.30]	0.44	[0.38, 0.51]	0.99	[0.98, 1.00]
Disg–fea						
30:70	0.39	[0.32, 0.46]	0.61	[0.54, 0.68]	0.99	[0.98, 1.00]
50:50	NA	NA	0.42	[0.36, 0.48]	0.97	[0.95, 0.99]
70:30	0.16	[0.11, 0.21]	0.26	[0.20, 0.32]	0.97	[0.95, 0.99]
Disg–hap						
30:70	0.45	[0.37, 0.53]	0.54	[0.46, 0.61]	0.99	[0.98, 1.00]
50:50	NA	NA	0.66	[0.58, 0.74]	0.98	[0.96, 0.99]
70:30	0.23	[0.17, 0.30]	0.38	[0.30, 0.47]	0.93	[0.90, 0.96]
Disg–sad						
30:70	0.24	[0.18, 0.30]	0.32	[0.26, 0.38]	0.99	[0.98, 1.00]
50:50	NA	NA	0.46	[0.40, 0.52]	1.00	[0.99, 1.00]
70:30	0.09	[0.053, 0.12]	0.29	[0.23, 0.35]	0.98	[0.96, 1.00]
Fea–hap						
30:70	0.41	[0.34, 0.47]	0.60	[0.53, 0.73]	0.98	[0.96, 1.00]
50:50	NA	NA	0.66	[0.58, 0.73]	0.98	[0.96, 1.00]
70:30	0.29	[0.23, 0.36]	0.41	[0.34, 0.49]	0.94	[0.90, 0.98]
Fea–sad						
30:70	0.53	[0.47, 0.60]	0.69	[0.63, 0.76]	1.00	[1.00, 1.00]
50:50	NA	NA	0.71	[0.65, 0.76]	1.00	[0.99, 1.00]
70:30	0.27	[0.21, 0.33]	0.60	[0.54, 0.66]	0.98	[0.96, 1.00]
Hap–sad						
30:70	0.22	[0.17, 0.28]	0.41	[0.36, 0.46]	0.97	[0.95, 1.00]
50:50	NA	NA	0.70	[0.63, 0.76]	0.97	[0.95, 0.99]
70:30	0.40	[0.33, 0.46]	0.72	[0.66, 0.78]	1.00	[0.99, 1.00]

*N* = 43. ang = anger, disg = disgust, fea = fear, hap = happiness, sad = sadness. 30:70 = The first emotion is intended to be less prominent than the second emotion. 50:50 = Both emotions are intended to be equally prominent. 70:30 = The first emotion is intended to be more prominent than the second emotion. Generous accuracy: chance level = 0.70. Strict accuracy: chance level = 0.10. Very strict accuracy: chance level = 0.05.

^a^Very strict accuracy was only calculated for the 30:70 and 70:30 conditions.

## Study 2

Results from Study 1 suggest that participants were able to accurately recognize proportions of blended emotions. However, in that study participants were instructed that they had to make judgments on 2 (out of 5) scales. It has been argued that perceivers are prone to use informed guessing strategies in forced-choice tasks^45,46^. Although some intended emotions may not have had clear signal characteristics for some pairwise emotion combinations, they may have still appeared to be well recognized after other response choices were eliminated. Also, we do not know if participants would spontaneously have reported ratings on two scales without being prompted to do so. It remains a possibility that they would have preferred to use only one scale, which would have precluded reporting of blended emotions^30,31^, or even to use more than two scales. In Study 2 we therefore replicate Study 1, but with an important methodological change, in order to try to control for these possible confounds. Participants in Study 2 will not be forced to give ratings on two scales, but are instead free to give ratings on any number of scales.

We report all manipulations, measures, and participant exclusions. All analyses were pre-registered (https://osf.io/zegc9), except when noted otherwise. Data and code are available on the Open Science Framework (https://osf.io/thu94).

### Method

#### Participants

Participants consisted of 39 adults (25 women) who were between 19 and 33 years (*M* = 22.1, *SD* = 3.2). They were recruited through advertisements on university campuses and received two movie tickets as compensation. Selection criteria were the same as in Study 1, with the added constraint that no participant should have taken part in the previous study. Sample size was determined apriori using G*Power^39^ and the same design and settings as in Study 1, which resulted in a recommended sample size of N = 39. The final sample size matched the planned sample size, and a post hoc sensitivity analysis thus indicated 95% power to detect effects of f = 0.25 or larger at alpha = 0.05. Informed consent was collected online from each participant, and all methods were performed in accordance with relevant guidelines and regulations and the Declaration of Helsinki, with approval from the Swedish Ethical Review Authority (decision no. 2021-00972). No participants were excluded after data collection.

#### Emotion perception task

The stimuli in the emotion perception task consisted of the same 180 videos as in Study 1, and contained expressions of 10 pairwise emotion combinations portrayed in varying proportions (i.e., 30:70, 50:50, and 70:30). The videos were presented in random order (different for each participant) and all participants judged all stimuli. Participants were instructed to rate how clearly they perceived different emotions on 5 scales (anger, disgust, fear, happiness, and sadness) ranging from 0 (emotion not at all prominent) to 10 (emotion very prominent). They were not prompted to use any specific number of scales. The minimum number that they could use was one—they had to make at least one rating in order to proceed to the next video—and the maximum number of scales they could use was 5. In other aspects, the experimental procedure was identical to the one in Study 1. The experiment was conducted online using PsychoPy^44^ on the Pavlovia platform.

### Results

#### Emotion ratings

Like in Study 1, emotion ratings were first analyzed using one-way repeated measures ANOVAs with intended emotion combination (10 levels: anger–disgust, anger–fear, anger–happiness, anger–sadness, disgust–fear, disgust–happiness, disgust–sadness, fear–happiness, fear–sadness, happiness–sadness) as within-subject factor. Separate ANOVAs were performed for the participants’ mean ratings on each rating scale (anger, fear, disgust, happiness, and sadness), and for each stimulus proportion (i.e., 30:70, 50:50, and 70:30). Table 4 details the results from the ANOVAs, which showed significant main effects (*p*s < .001) demonstrating that ratings varied systematically as a function of emotion combination, for all rating scales in all stimulus proportion conditions.

**Table 4:** F-Statistics and effect size estimates for all emotion scales and proportion combinations (Study 2).

Emotion scale [emotion proportion]	*F*(9, 342)	*P*	η^2^_*p*_	η^2^_*G*_
Anger				
[30:70]	168.02	< .001	0.82	0.74
[50:50]	310.62	< .001	0.89	0.84
[70:30]	442.58	< .001	0.92	0.88
Disgust				
[30:70]	242.15	< .001	0.86	0.82
[50:50]	196.99	< .001	0.84	0.78
[70:30]	294.48	< .001	0.89	0.84
Fear				
[30:70]	150.54	< .001	0.80	0.71
[50:50]	105.16	< .001	0.74	0.64
[70:30]	138.72	< .001	0.78	0.70
Happiness				
[30:70]	443.26	< .001	0.92	0.89
[50:50]	252.91	< .001	0.87	0.81
[70:30]	149.27	< .001	0.80	0.72
Sadness				
[30:70]	431.94	< .001	0.92	0.88
[50:50]	167.54	< .001	0.81	0.74
[70:30]	141.72	< .001	0.79	0.71

*N* = 39. Mauchly’s test of sphericity showed that none of the emotion scales met the assumption of sphericity, but effects remained statistically significantly after Greenhouse–Geisser correction (all corrected *ps* < 0.0001). We used generalized eta squared (η^2^_*G*_) to estimate the variation explained by the within-subjects factor while accounting for both number of factors and individual differences (which enables comparison across studies regardless of design, and partial eta squared (η^2^_*p*_) to estimate the amount of variation explained by the within-subjects factor when controlling for the number of factors.

The main effects were followed up with post-hoc multiple comparisons (repeated measures *t*-tests) showing that all target emotion combinations received significantly higher ratings than the non-target combinations for the anger, disgust, happiness and sadness scales, for all stimulus proportions (*p*s < .0001, Bonferroni corrected alpha level = 0.0021, i.e. corrected for 24 comparisons per ANOVA). However, like in Study 1, some comparisons were not significant for the fear scale. In the 50:50 condition, the target combination 50:50 anger–fear (M = 2.12) was not rated significantly higher on fear than the non-target combination 50:50 disgust–sadness (M = 1.84), see Table 5. In the 70:30 condition, the target combinations 70:30 disgust–fear (M = 0.94) and 70:30 anger–fear (M = 1.91) were both rated lower on the fear scale (instead of higher) than the non-target combinations 70:30 disgust–happiness (M = 2.03) and 70:30 disgust–sadness (M = 2.31). The fear rating for 70:30 disgust–fear was in fact so low that it was not significantly higher than any non-target combination. The comparisons between target and non-target combinations are presented in full in Table S3.

**Table 5:** Emotion ratings for each emotion combination and emotion proportion (Study 2).

Emotion combination	Emotion ratings (M/SD)				
	Anger	Disgust	Fear	Happiness	Sadness
Ang–disg					
30:70*	**3.13 (1.14)**	**6.76 (1.24)**	0.73 (0.65)	0.21 (0.65)	0.50 (0.83)
50:50	**4.89 (1.25)**	**4.89 (1.27)**	0.68 (0.72)	0.10 (0.61)	0.37 (0.62)
70:30*	**5.90 (1.13)**	**3.15 (1.02)**	0.48 (0.71)	0.03 (0.64)	0.46 (0.74)
Ang–fea					
30:70	**4.73 (1.18)**	0.59 (0.73)	**3.62 (1.24)**	0.06 (0.61)	1.94 (0.93)
50:50	**7.54 (1.50)**	0.35 (0.59)	**2.12 (1.23)**	0.11 (0.65)	0.68 (0.72)
70:30*	**7.74 (1.07)**	0.73 (0.68)	**1.91 (1.15)**	0.01 (0.66)	0.20 (0.71)
Ang–hap					
30:70*	**2.53 (0.96)**	0.28 (0.56)	0.31 (0.66)	**7.15 (1.45)**	0.16 (0.61)
50:50	**4.68 (1.43)**	0.40 (0.69)	0.23 (0.70)	**5.40 (1.38)**	0.13 (0.68)
70:30*	**5.80 (0.99)**	0.56 (0.72)	0.87 (0.62)	**2.89 (1.16)**	0.25 (0.58)
Ang–sad					
30:70*	**5.04 (1.16)**	0.36 (0.63)	1.08 (0.93)	0.21 (0.64)	**6.57 (1.30)**
50:50	**6.59 (1.14)**	0.56 (0.91)	1.20 (0.74)	0.43 (0.66)	**4.18 (1.35)**
70:30*	**6.45 (1.03)**	0.63 (0.82)	1.06 (0.83)	0.49 (0.64)	**2.39 (1.09)**
Disg–fea					
30:70*	0.88 (0.72)	**3.89 (1.07)**	**6.03 (1.11)**	0.05 (0.64)	0.71 (0.75)
50:50	1.63 (0.62)	**6.06 (1.44)**	**2.91 (1.06)**	0.29 (0.65)	1.32 (0.73)
70:30*	1.97 (1.03)	**7.36 (1.45)**	**0.94 (0.86)**	0.18 (0.62)	0.68 (0.68)
Disg–hap					
30:70*	0.41 (0.74)	**2.25 (1.09)**	0.39 (0.58)	**7.40 (1.34)**	0.64 (0.67)
50:50	0.26 (0.69)	**4.71 (1.38)**	0.59 (0.64)	**6.17 (1.99)**	0.53 (0.90)
70:30*	0.40 (0.75)	**6.50 (1.50)**	2.03 (1.03)	**2.98 (1.47)**	0.50 (0.67)
Disg–sad					
30:70*	0.90 (0.66)	**1.41 (0.81)**	0.91 (0.88)	0.09 (0.62)	**7.41 (1.10)**
50:50	0.91 (0.76)	**4.60 (1.65)**	1.84 (0.86)	0.20 (0.71)	**4.62 (1.32)**
70:30	0.89 (0.91)	**3.71 (0.80)**	2.31 (0.77)	0.08 (0.70)	**3.58 (0.85)**
Fea–hap					
30:70*	0.34 (0.80)	0.38 (0.74)	**2.10 (1.10)**	**6.86 (1.56)**	0.27 (0.83)
50:50	0.16 (0.64)	0.56 (0.61)	**3.80 (1.33)**	**5.62 (1.10)**	0.37 (0.70)
70:30*	0.10 (0.62)	0.85 (0.78)	**5.17 (1.31)**	**2.85 (1.03)**	1.09 (0.78)
Fea–sad					
30:70*	0.38 (0.68)	0.09 (0.62)	**2.42 (0.99)**	0.00 (0.67)	**7.97 (1.18)**
50:50	0.29 (0.70)	0.55 (0.64)	**5.11 (1.49)**	0.08 (0.63)	**5.47 (1.54)**
70:30*	0.36 (0.75)	0.56 (0.74)	**5.47 (1.18)**	0.21 (0.67)	**3.65 (0.91)**
Hap–sad					
30:70*	0.20 (0.69)	0.47 (0.81)	0.81 (0.75)	**2.11 (0.80)**	**6.50 (1.43)**
50:50	0.44 (0.80)	0.21 (0.72)	0.24 (0.59)	**5.24 (1.38)**	**4.61 (1.25)**
70:30	0.16 (0.68)	0.11 (0.53)	0.67 (0.70)	**5.40 (1.38)**	**4.25 (1.24)**

*N* = 39. ang = anger, disg = disgust, fea = fear, hap = happiness, sad = sadness. 30:70 = The first emotion is intended to be less prominent than the second emotion. 50:50 = Both emotions are intended to be equally prominent. 70:30 = The first emotion is intended to be more prominent than the second emotion. Values in bold indicate emotion combinations that contain emotions that correspond to the rating scale (i.e., target combinations). Asterisks indicate emotion combinations where proportions between emotions were accurately judged (i.e., significantly higher ratings for the more vs. the less prominent emotion) as indicated by post-hoc repeated measures *t*-tests, *p*s < .0025. Emotion ratings: 0 = Emotion not at all prominent, 10 = Emotion very prominent.

##### Exploratory analyses

Like in Study 1, we also carried out post-hoc multiple comparisons (repeated measures *t*-tests with alpha = 0.0025, i.e., corrected for 20 comparisons) to investigate if the proportions in the 30:70 and 70:30 conditions were accurately perceived. Similar to Study 1, the majority of tests showed that the more prominent emotion in each pair received significantly higher ratings than the less prominent emotion, see Table 5. The only exceptions were observed for 70:30 disgust–sadness where the difference between disgust ratings (M = 3.71) and sadness ratings (M = 3.58) was not significant (*p* = .593), and for 70:30 happiness–sadness where the difference between happiness (M = 5.40) and sadness (M = 4.25) ratings was not significant (*p* = .003). All details from the exploratory analyses are shown in Table S4.

**Table 6:** Emotion recognition accuracy for each emotion combination and emotion proportion (Study 2).

Emotion combination	Emotion recognition accuracy					
	Very strict ^a^	95% CI	Strict	95% CI	Generous	95% CI
Ang–disg						
30:70	0.29	[0.23, 0.35]	0.46	[0.39, 0.53]	0.91	[0.88, 0.94]
50:50	NA	NA	0.54	[0.46, 0.62]	0.95	[0.93, 0.98]
70:30	0.25	[0.20, 0.30]	0.44	[0.38, 0.51]	0.93	[0.89, 0.96]
Ang–fea						
30:70	0.06	[0.03, 0.09]	0.29	[0.23, 0.35]	0.76	[0.70, 0.81]
50:50	NA	NA	0.38	[0.30, 0.46]	0.95	[0.92, 0.99]
70:30	0.24	[0.17, 0.30]	0.32	[0.24, 0.40]	0.98	[0.97, 1.00]
Ang–hap						
30:70	0.41	[0.32, 0.50]	0.53	[0.44, 0.61]	0.97	[0.94, 1.00]
50:50	NA	NA	0.70	[0.62, 0.78]	0.95	[0.93, 0.98]
70:30	0.34	[0.27, 0.40]	0.47	[0.38, 0.56]	0.88	[0.85, 0.92]
Ang–sad						
30:70	0.29	[0.23, 0.35]	0.65	[0.59, 0.71]	0.91	[0.87, 0.96]
50:50	NA	NA	0.57	[0.48, 0.65]	0.90	[0.85, 0.94]
70:30	0.13	[0.08, 0.18]	0.28	[0.22, 0.35]	0.86	[0.82, 0.91]
Disg–fea						
30:70	0.33	[0.26, 0.40]	0.44	[0.37, 0.51]	0.91	[0.87, 0.95]
50:50	NA	NA	0.34	[0.28, 0.39]	0.73	[0.67, 0.78]
70:30	0.13	[0.08, 0.19]	0.16	[0.104, 0.21]	0.81	[0.76, 0.87]
Disg–hap						
30:70	0.45	[0.38, 0.52]	0.52	[0.46, 0.58]	0.89	[0.85, 0.93]
50:50	NA	NA	0.69	[0.61, 0.77]	0.90	[0.86, 0.94]
70:30	0.21	[0.16, 0.26]	0.37	[0.30, 0.44]	0.78	[0.72, 0.85]
Disg–sad						
30:70	0.18	[0.12, 0.24]	0.21	[0.15, 0.28]	0.91	[0.87, 0.95]
50:50	NA	NA	0.38	[0.30, 0.46]	0.78	[0.73, 0.83]
70:30	0.06	[0.02, 0.09]	0.24	[0.19, 0.30]	0.73	[0.69, 0.77]
Fea–hap						
30:70	0.42	[0.33, 0.51]	0.50	[0.41, 0.58]	0.94	[0.90, 0.98]
50:50	NA	NA	0.59	[0.50, 0.67]	0.93	[0.89, 0.97]
70:30	0.29	[0.23, 0.35]	0.46	[0.38, 0.53]	0.77	[0.73, 0.81]
Fea–sad						
30:70	0.31	[0.23, 0.39]	0.41	[0.33, 0.49]	1.00	[0.99, 1.00]
50:50	NA	NA	0.59	[0.50, 0.68]	0.95	[0.93, 0.98]
70:30	0.17	[0.12, 0.21]	0.44	[0.37, 0.51]	0.94	[0.90, 0.98]
Hap–sad						
30:70	0.18	[0.13, 0.23]	0.33	[0.27, 0.39]	0.92	[0.87, 0.96]
50:50	NA	NA	0.65	[0.56, 0.73]	0.93	[0.89, 0.96]
70:30	0.36	[0.29, 0.43]	0.63	[0.54, 0.71]	0.93	[0.88, 0.97]

*N* = 39. ang = anger, disg = disgust, fea = fear, hap = happiness, sad = sadness. 30:70 = The first emotion is intended to be less prominent than the second emotion. 50:50 = Both emotions are intended to be equally prominent. 70:30 = The first emotion is intended to be more prominent than the second emotion. Generous accuracy: approximate chance level = 0.70. Strict accuracy: approximate chance level = 0.10. Very strict accuracy: approximate chance level = 0.05.

^a^Very strict accuracy was only calculated for the 30:70 and 70:30 conditions.

Because the participants were free to use any number of scales (between 1 and 5), we further investigated how they chose to distribute their answers. The average number of scales used was 1.95 (SD = 0.71), which corresponded well with the number of intended emotions in the pairwise combinations. The majority of responses consisted of ratings on two scales (56.98%), but 25.26% of the responses consisted of ratings on one scale only. Responses with ratings on three scales were also relatively common (15.66%), whereas responses on four (1.95%) and five (0.16%) scales were scarce. Table S5 contains a breakdown of the frequencies of responses on different numbers of scales for all conditions.

#### Accuracy indices

Like in Study 1, emotion ratings were also used to calculate measures of the extent to which participants recognized one of the two intended emotions (generous accuracy), both of the two intended emotions (strict accuracy), and both of the intended emotions as well as their proportions (very strict accuracy). It should be noted that chance levels in Study 2 vary depending on how many response options participants selected on each trial, making it impossible to define a general chance level for each accuracy index. However, because most responses involved two or fewer selected emotions, we applied the same chance levels as in Study 1 as a general reference point for assessing performance. This should be interpreted with caution, as trials involving more than two selected emotions inherently have higher chance levels. Overall strict accuracy (M = 0.45, SD = 0.50, 95% CI [0.44, 0.46]) and overall very strict accuracy (M = 0.25, SD = 0.44, 95% CI [0.24, 0.27]) were both above chance, as indicated by confidence intervals that did not overlap with the corresponding approximate chance levels. Table 6 presents the accuracy indices for all emotion combinations for each emotion proportion condition (note that very strict accuracy only applies to the 30:70 and 70:30 conditions). All three accuracy indices were higher than what would be expected by chance guessing for nearly all emotion combinations, as indicated by 95% CIs not overlapping the respective approximate chance level. Two notable exceptions were observed for very strict accuracy for 30:70 anger–fear (M = 0.06, 95% CI [0.03, 0.09]), and, like in Study 1, for 70:30 disgust–sadness (M = 0.06, 95% CI [0.02, 0.09]).

## Discussion

We asked actors to express blended emotions consisting of pairwise combinations of anger, disgust, fear, happiness, and sadness through dynamic multimodal (facial, vocal, and bodily) expressions. The two emotions in each pairwise combination were portrayed with different proportions, either equally prominent or differentially prominent (with one emotion more prominent than the other). The portrayals were assessed in two preregistered studies using variations of a combined rating scale and forced-choice task, which enabled us to analyze both ratings and accuracy measures. Results were quite consistent across studies and analyses, and first showed that all blended emotions received significantly higher ratings on scales corresponding to intended vs. non-intended emotions. Measures of accuracy (strict accuracy) also indicated that participants were able to recognize both of the intended emotions from each emotion combination with above-chance accuracy. These findings replicate previous studies—most directly Israelsson et al.^14^ but also Nummenmaa^22,23^ and Pallett and Martinez^26^—which suggested that individuals can accurately perceive pairwise combinations of emotions where both emotions are equally prominent. Our findings also extend previous research by showing that pairwise combinations can be accurately perceived when one emotion in the pair is expressed more prominently than the other.

In a novel approach, we further directly investigated if individuals were able to correctly judge the different proportions of emotions. Again, results were quite consistent across studies and showed that the more prominent emotion of the pair usually received higher ratings than the less prominent emotion, on their respective scales. Measures of accuracy (very strict accuracy) confirmed that both of the two intended emotions as well as their proportions were usually recognized with above-chance accuracy. In summary, our findings suggest that it is possible to express and perceive both the presence (i.e., which emotions are included in the blend) and the relative salience (i.e., how much of each emotion is included in the blend) from dynamic multimodal expressions of blended emotions.

Recognition rates were in general significantly lower in Study 2 than in Study 1, as indicated by non-overlapping confidence intervals between studies for both the strict and very strict indices of overall accuracy. This suggests that accuracy may have been affected by the fact that participants were prompted to use two scales in Study 1 whereas they were free to use any number of scales in Study 2. For example, participants in Study 1 may have used some informed guessing strategies to determine the second emotion^45,46^. This interpretation is tentative, as differences between the two studies may also reflect possible group differences in emotion recognition ability and related skills, rather than just task differences. However, the groups were comparable in age and gender composition and were recruited from similar populations. It should be noted that strict accuracy was mostly above chance also in Study 2, which shows that blended emotions were accurately recognized also when participants were not prompted to provide ratings on two scales^31^. Our analysis of scale usage in Study 2 further showed that a majority of responses did provide ratings on two scales, which indicates that participants often spontaneously perceived two emotions. Around 25% of the responses contained ratings on only one scale, however, and 16% contained ratings on three scales, which in turn indicates that participants did not always spontaneously perceive and report exactly two emotions. Nevertheless, results were quite consistent across rating tasks, which indicates that most emotion combinations have signal characteristics that can be picked up by perceivers regardless of how many scales were used in the rating task.

The main pattern was that participants generally recognized the intended blended emotions with above-chance recognition accuracy, but some emotion combinations showed weaker differentiation in scale ratings. In particular, across both studies, combinations that included fear were harder to recognize. This could indicate that fear may be particularly hard to detect when combined with other emotions such as anger and disgust. This may have been caused by expressive features associated with fear being overshadowed by more prominent ones associated with other intended emotions with similar levels of arousal and valence^30^, and consequently being harder to detect. However, results also showed that participants sometimes reported fear in emotion combinations where fear was not an intended emotion, which in turn could indicate that features associated with fear were present also in such combinations. These observations are in line with previous work suggesting that observers may perceive multiple emotions from single-emotion expressions and that non-intended emotions are more likely to be endorsed when they are morphologically similar to the intended ones^17,21^.

Our study did not investigate how blended emotions were expressed through various expressive features, which is an important objective for future research. Methodological development related to analysis of facial and vocal features has seen rapid progress^47^, and we argue that sophisticated feature extraction could be coupled with explainable machine learning methods^48^ to investigate in detail how blended emotions are conveyed through complex patterns of features that evolve over the course of a dynamic emotion expression^3,49^. A better understanding of how blended emotions are expressed—whether by rapid shifts between expressions, combinations of features from different emotions (e.g., a sad mouth with happy eyes), or unique patterns not seen in single emotions—is essential for advancing the science of nonverbal behavior. Such data could help refine theories about the subjective experience of blended and complex emotions^11,13,50^. It may also be used to evaluate competing accounts of how emotional expressions are generated and how they relate to other components of the emotion process, such as appraisal, physiological arousal or subjective experience^51^.

### Limitations

Several methodological limitations should be taken into account when interpreting the findings of this study. We start by noting that the present results are based on actor portrayals, which may differ from naturalistic expressions of blended emotions. Evidence from studies on single emotions suggest that actor portrayals and spontaneous expressions may differ in subtle but systematic ways^52–54^. Creating a multimodal database of dynamic naturalistic expressions of blended emotions is an ambitious and interesting objective for future research. Another methodological consideration concerns the use of relative rather than quantitatively specified emotion proportions in the stimulus material. Actors were instructed to express one emotion as less prominent, equally prominent, or more prominent than the other, rather than being constrained to exact proportions. This approach was chosen to facilitate the acting process and to increase the naturalistic quality of the expressions, but may also limit precise experimental control over emotion proportions. Nevertheless, participants’ ratings varied systematically across proportion conditions, indicating that the intended differences in relative salience were reliably perceived. However, it remains to be determined whether these perceptual differences are directly associated with specific expressive characteristics, as no feature-level analyses were conducted in the present study.

The forced-choice response format used in the perception tasks presents another limitation. As noted, perceivers may rely on informed guessing in forced-choice tasks^45,46^. As a result, even when a particular emotion lacks clear distinguishing features, it may still appear well recognized because other response alternatives can be excluded. In addition, although we systematically investigated the effect of prompting vs. not prompting the number of scales to use, our tasks did not allow for responses outside of the given scales. Future studies could use open ended tasks^55^ to further investigate how blended emotions are spontaneously perceived. Such methods could be used to test theoretical propositions about how blending two emotions may create a new emotion that is distinct from the two original emotions^56^.

Participants were allowed to view the video clips as many times as they wished, ensuring that their judgments reflected perceived information rather than guesses due to inattention. However, this approach may somewhat reduce ecological validity, as emotional expressions in real-life interactions are often brief and cannot be revisited. Furthermore, the online data collection format did not allow full control of participants’ viewing distance, screen characteristics, audio equipment, or listening environment. Although participation was restricted to computers and stimulus size was standardized to ensure consistency across screen resolutions, and participants were instructed to use headphones, these factors remain potential sources of variability and should be considered when interpreting the results. Finally, while our pre-registered analyses employed a fully balanced design with no missing data and adjustments for multiple comparisons, we acknowledge that the data has a nested structure (e.g., trials within participants). Future research could employ analysis strategies such as multilevel modeling to more explicitly account for the random effects of both participants and stimuli.

### Conclusions

To summarize, our findings suggest that when individuals are presented with actor portrayals of dynamic multimodal expressions combining two emotions in different proportions, they are generally able to accurately judge both which emotions, and how much of each, was intended. Although this ability may vary slightly across specific emotion combinations, as a whole our results reveal nuanced nonverbal communication of both the presence and relative salience of blended emotions, which may help us navigate in a complex social environment where blended emotions are not uncommon^13^.

## Supplementary Information

Supplementary Information.

## Data Availability

All analyses were pre-registered [https://osf.io/ngazb (Study 1); https://osf.io/zegc9 (Study 2)], except when noted otherwise. Data and code are available on the Open Science Framework [https://osf.io/pws8u Study 1); https://osf.io/thu94 (Study 2)]. All measures, manipulations, and participant exclusions are reported in this article or its Supplementary Materials for both Study 1 and Study 2.

## Declarations

### Open practices

All analyses were pre-registered [https://osf.io/ngazb (Study 1); https://osf.io/zegc9 (Study 2)], except when noted otherwise. Data and code are available on the Open Science Framework [https://osf.io/pws8u Study 1); https://osf.io/thu94 (Study 2)]. All measures, manipulations, and participant exclusions are reported in this article or its Supplementary Materials for both Study 1 and Study 2.
