# Supplementary material for: Dynamic multimodal expressions support inference of both presence and relative salience of blended emotions
Source: Sci Rep. 2026 Jun 6;16:17536. doi: 10.1038/s41598-026-55904-z (PMC13242507; doi:10.1038/s41598-026-55904-z)
Supplement: Supplementary file 1 — Supplementary Information. [file 41598_2026_55904_MOESM1_ESM.docx]

## Supplementary Information

Israelsson, A., Sandberg, G., Zeitler, S., & Laukka, P. (2026). *Dynamic multimodal expressions support inference of both presence and relative salience of blended emotions.* Manuscript submitted for publication.

## The supplementary information contains 5 tables (Table S1–S5).

## Table S1

*Multiple Pairwise Comparisons Between Intended and Non-intended Emotion Blends for Respective Emotion Scale (Study 1).*

| Anger |  | | | | | | | | | | | | | | | | | | |
| --- | --- | --- | --- | --- | --- | --- | --- | --- | --- | --- | --- | --- | --- | --- | --- | --- | --- | --- | --- |
|  | Intended | | | | | | | | | | | | | | | | | | |
|  | Ang-disg | | | |  | Ang-fea | | | |  | Ang-hap | | | |  | Ang-sad | | | |
| Non- intended | *t*(42) | *M* ^diff^ | *SE* | CI 95% |  | *t*(42) | *M* ^diff^ | *SE* | CI 95% |  | *t*(42) | *M* ^diff^ | *SE* | CI 95% |  | *t*(42) | *M* ^diff^ | *SE* | CI 95% |
| **Disg-fea** |  |  |  |  |  |  |  |  |  |  |  |  |  |  |  |  |  |  |  |
| [30:70] | 15.64 | 2.91* | 0.19 | [2.54, 3.29] |  | 20.92 | 4.21* | 0.20 | [3.81, 4.62] |  | 11.78 | 2.22* | 0.19 | [1.84, 2.61] |  | 15.94 | 4.41* | 0.28 | [3.85, 4.97] |
| [50:50] | 12.42 | 3.21* | 0.26 | [2.69, 3.74] |  | 23.47 | 6.26* | 0.27 | [5.72, 6.80] |  | 11.28 | 3.34* | 0.30 | [2.74, 3.93] |  | 23.98 | 5.15* | 0.21 | [4.71, 5.58] |
| [70:30] | 18.93 | 3.98* | 0.21 | [3.55, 4.40] |  | 31.45 | 6.21* | 0.20 | [5.81, 6.61] |  | 16.78 | 3.83* | 0.23 | [3.37, 4.29] |  | 20.16 | 4.29* | 0.21 | [3.86, 4.72] |
| **Disg-hap** |  |  |  |  |  |  |  |  |  |  |  |  |  |  |  |  |  |  |  |
| [30:70] | 15.40 | 3.38* | 0.22 | [2.93, 3.82] |  | 20.03 | 4.67* | 0.23 | [4.20, 5.15] |  | 13.94 | 2.69* | 0.19 | [2.30, 3.07] |  | 15.87 | 4.87* | 0.31 | [4.25, 5.49] |
| [50:50] | 18.04 | 4.60* | 0.25 | [4.08, 5.11] |  | 27.31 | 7.64* | 0.28 | [7.08, 8.21] |  | 14.78 | 4.72* | 0.32 | [4.08, 5.37] |  | 27.44 | 6.53* | 0.24 | [6.05, 7.01] |
| [70:30] | 20.81 | 5.31* | 0.25 | [4.79, 5.82] |  | 33.90 | 7.54* | 0.22 | [7.09, 7.99] |  | 19.32 | 5.16* | 0.27 | [4.62, 5.70] |  | 24.15 | 5.62* | 0.23 | [5.15, 6.09] |
| **Disg-sad** |  |  |  |  |  |  |  |  |  |  |  |  |  |  |  |  |  |  |  |
| [30:70] | 15.06 | 2.78* | 0.18 | [2.41, 3.15] |  | 20.57 | 4.08* | 0.20 | [3.68, 4.48] |  | 12.52 | 2.09* | 0.17 | [1.75, 2.43] |  | 16.04 | 4.27* | 0.27 | [3.73, 4.81] |
| [50:50] | 16.21 | 4.03* | 0.25 | [3.53, 4.53] |  | 25.79 | 7.08* | 0.27 | [6.52, 7.63] |  | 14.47 | 4.16* | 0.29 | [3.58, 4.73] |  | 27.35 | 5.97* | 0.22 | [5.52, 6.41] |
| [70:30] | 22.64 | 5.35* | 0.24 | [4.87, 5.83] |  | 38.32 | 7.58* | 0.20 | [7.18, 7.98] |  | 22.84 | 5.21* | 0.23 | [4.75, 5.67] |  | 30.47 | 5.66* | 0.19 | [5.28, 6.03] |
| **Table S1 (Continued)** |  | | | | | | | | | | | | | | | | | | |
| Anger (continue) |  | | | | | | | | | | | | | | | | | | |
|  | Intended | | | | | | | | | | | | | | | | | | |
|  | Ang-disg | | | |  | Ang-fea | | | |  | Ang-hap | | | |  | Ang-sad | | | |
| Non- intended | *t*(42) | *M* ^diff^ | *SE* | CI 95% |  | *t*(42) | *M* ^diff^ | *SE* | CI 95% |  | *t*(42) | *M* ^diff^ | *SE* | CI 95% |  | *t*(42) | *M* ^diff^ | *SE* | CI 95% |
| **Fea-hap** |  |  |  |  |  |  |  |  |  |  |  |  |  |  |  |  |  |  |  |
| [30:70] | 13.47 | 3.06* | 0.23 | [2.60, 3.52] |  | 16.86 | 4.36* | 0.26 | [3.83, 4.88] |  | 13.30 | 2.37* | 0.18 | [2.01, 2.73] |  | 14.16 | 4.55* | 0.32 | [3.90, 5.20] |
| [50:50] | 17.66 | 4.60* | 0.26 | [4.07, 5.12] |  | 31.08 | 7.64* | 0.25 | [7.15, 8.14] |  | 15.26 | 4.72* | 0.31 | [4.10, 5.35] |  | 28.50 | 6.53* | 0.23 | [6.07, 6.99] |
| [70:30] | 26.66 | 5.86* | 0.22 | [5.41, 6.30] |  | 43.77 | 8.09* | 0.18 | [7.72, 8.46] |  | 26.76 | 5.71* | 0.21 | [5.28, 6.14] |  | 36.94 | 6.17* | 0.17 | [5.83, 6.50] |
| **Fea-sad** |  |  |  |  |  |  |  |  |  |  |  |  |  |  |  |  |  |  |  |
| [30:70] | 14.08 | 3.07* | 0.22 | [2.63, 3.51] |  | 22.69 | 4.36* | 0.19 | [3.98, 4.75] |  | 13.66 | 2.38* | 0.17 | [2.02, 2.73] |  | 15.70 | 4.56* | 0.29 | [3.97, 5.14] |
| [50:50] | 16.94 | 4.41* | 0.26 | [3.89, 4.94] |  | 34.18 | 7.46* | 0.22 | [7.02, 7.90] |  | 15.18 | 4.53* | 0.30 | [3.93, 5.14] |  | 26.98 | 6.34* | 0.24 | [5.87, 6.82] |
| [70:30] | 26.13 | 5.80* | 0.22 | [5.35, 6.25] |  | 42.38 | 8.03* | 0.19 | [7.65, 8.42] |  | 28.38 | 5.66* | 0.20 | [5.26, 6.06] |  | 35.76 | 6.11* | 0.17 | [5.77, 6.46] |
| **Hap-sad** |  |  |  |  |  |  |  |  |  |  |  |  |  |  |  |  |  |  |  |
| [30:70] | 17.25 | 3.32* | 0.19 | [2.93, 3.71] |  | 19.70 | 4.62* | 0.23 | [4.14, 5.09] |  | 15.30 | 2.63* | 0.17 | [2.28, 2.97] |  | 16.10 | 4.81* | 0.30 | [4.21, 5.41] |
| [50:50] | 18.17 | 4.48* | 0.25 | [3.98, 4.98] |  | 31.59 | 7.53* | 0.24 | [7.05, 8.01] |  | 15.85 | 4.60* | 0.29 | [4.02, 5.19] |  | 29.27 | 6.41* | 0.22 | [5.97, 6.86] |
| [70:30] | 24.98 | 5.93* | 0.24 | [5.45, 6.41] |  | 41.24 | 8.16* | 0.20 | [7.76, 8.56] |  | 27.04 | 5.79* | 0.21 | [5.35, 6.22] |  | 36.12 | 6.24* | 0.17 | [5.89, 6.59] |
|  |  |  |  |  |  |  |  |  |  |  |  |  |  |  |  |  |  |  |  |

| **Table S1 (Continued)** | | |  |  |  |  |  |  |  |  |  |  |  |  |  |  |  |  |  |
| --- | --- | --- | --- | --- | --- | --- | --- | --- | --- | --- | --- | --- | --- | --- | --- | --- | --- | --- | --- |
| Disgust |  |  |  |  |  |  |  |  |  |  |  |  |  |  |  |  |  |  |  |
|  | Intended | | | | | | | | | | | | | | | | | | |
|  | Ang-disg | | | |  | Disg-fea | | | |  | Disg-hap | | | |  | Disg-sad | | | |
| Non- intended | *t*(42) | *M* ^diff^ | *SE* | CI 95% |  | *t*(42) | *M* ^diff^ | *SE* | CI 95% |  | *t*(42) | *M* ^diff^ | *SE* | CI 95% |  | *t*(42) | *M* ^diff^ | *SE* | CI 95% |
| **Ang-fea** |  |  |  |  |  |  |  |  |  |  |  |  |  |  |  |  |  |  |  |
| [30:70] | 21.02 | 6.09* | 0.29 | [5.50, 6.67] |  | 13.16 | 3.54* | 0.27 | [3.00, 4.08] |  | 4.87 | 1.39* | 0.29 | [0.82, 1.97] |  | 3.96 | 0.85* | 0.21 | [0.42, 1.28] |
| [50:50] | 18.95 | 4.53* | 0.24 | [4.05, 5.02] |  | 22.82 | 5.64* | 0.25 | [5.14, 6.13] |  | 11.83 | 3.60* | 0.30 | [2.98, 4.21] |  | 15.07 | 3.64* | 0.24 | [3.16, 4.13] |
| [70:30] | 10.27 | 2.58* | 0.25 | [2.07, 3.09] |  | 22.21 | 6.28* | 0.28 | [5.70, 6.85] |  | 16.91 | 5.09* | 0.30 | [4.49, 5.70] |  | 14.27 | 3.28* | 0.23 | [2.82, 3.74] |
| **Ang-hap** |  |  |  |  |  |  |  |  |  |  |  |  |  |  |  |  |  |  |  |
| [30:70] | 25.99 | 6.71* | 0.26 | [6.18, 7.23] |  | 14.96 | 4.16* | 0.28 | [3.59, 4.72] |  | 9.10 | 2.01* | 0.22 | [1.56, 2.45] |  | 7.36 | 1.47* | 0.20 | [1,06, 1.87] |
| [50:50] | 18.02 | 4.88* | 0.27 | [4.33, 5.42] |  | 21.79 | 5.98* | 0.27 | [5.42, 6.53] |  | 12.76 | 3.94* | 0.31 | [3.31, 4.56] |  | 14.60 | 3.98* | 0.27 | [3.43, 4.54] |
| [70:30] | 12.21 | 2.91* | 0.24 | [2.43, 3.40] |  | 21.13 | 6.61* | 0.31 | [5.98, 7.24] |  | 15.88 | 5.43* | 0.34 | [4.74, 6.12] |  | 14.16 | 3.61* | 0.26 | [3.10, 4.13] |
| **Ang-sad** |  |  |  |  |  |  |  |  |  |  |  |  |  |  |  |  |  |  |  |
| [30:70] | 25.56 | 6.65* | 0.26 | [6.13, 7.18] |  | 16.54 | 4.10* | 0.25 | [3.60, 4.60] |  | 7.61 | 1.95* | 0.26 | [1.44, 2.47] |  | 6.94 | 1.41* | 0.20 | [1.00, 1.82] |
| [50:50] | 19.68 | 4.81* | 0.24 | [4.31, 5.30] |  | 24.67 | 5.91* | 0.24 | [5.42, 6.39] |  | 13.96 | 3.87* | 0.28 | [3.31, 4.43] |  | 15.50 | 3.91* | 0.25 | [3.41, 4.42] |
| [70:30] | 12.02 | 2.78* | 0.23 | [2.31, 3.25] |  | 22.18 | 6.47* | 0.29 | [5.88, 7.06] |  | 18.12 | 5.29* | 0.29 | [4.70, 5.88] |  | 15.00 | 3.48* | 0.23 | [3.01, 3.94] |
|  |  |  |  |  |  |  |  |  |  |  |  |  |  |  |  |  |  |  |  |
|  |  |  |  |  |  |  |  |  |  |  |  |  |  |  |  |  |  |  |  |
| **Table S1 (Continued)** | | |  |  |  |  |  |  |  |  |  |  |  |  |  |  |  |  |  |
| Disgust  (continue) |  |  |  |  |  |  |  |  |  |  |  |  |  |  |  |  |  |  |  |
|  | Intended | | | | | | | | | | | | | | | | | | |
|  | Ang-disg | | | |  | Disg-fea | | | |  | Disg-hap | | | |  | Disg-sad | | | |
| Non- intended | *t*(42) | *M* ^diff^ | *SE* | CI 95% |  | *t*(42) | *M* ^diff^ | *SE* | CI 95% |  | *t*(42) | *M* ^diff^ | *SE* | CI 95% |  | *t*(42) | *M* ^diff^ | *SE* | CI 95% |
| **Fea-hap** |  |  |  |  |  |  |  |  |  |  |  |  |  |  |  |  |  |  |  |
| [30:70] | 27.86 | 6.74* | 0.24 | [6.26, 7.23] |  | 15.95 | 4.19* | 0.26 | [3.66, 4.72] |  | 9.17 | 2.05* | 0.22 | [1.60, 2.50] |  | 8.25 | 1.50* | 0.18 | [1.14, 1.87] |
| [50:50] | 18.15 | 4.80* | 0.26 | [4.27, 5.33] |  | 25.43 | 5.90* | 0.23 | [5.43, 6.37] |  | 15.84 | 3.86* | 0.24 | [3.37, 4.35] |  | 16.86 | 3.91* | 0.23 | [3.44, 4.37] |
| [70:30] | 11.14 | 2.24* | 0.20 | [1.83, 2.65] |  | 20.67 | 5.93* | 0.29 | [5.35, 6.51] |  | 17.61 | 4.75* | 0.27 | [4.21, 5.30] |  | 15.43 | 2.94* | 0.19 | [2.55, 3.32] |
| **Fea-sad** |  |  |  |  |  |  |  |  |  |  |  |  |  |  |  |  |  |  |  |
| [30:70] | 25.30 | 6.61* | 0.26 | [6.09, 7.14] |  | 16.32 | 4.06* | 0.25 | [3.56, 4.56] |  | 7.97 | 1.91* | 0.24 | [1.43, 2.40] |  | 9.13 | 1.37* | 0.15 | [1.07, 1.68] |
| [50:50] | 19.09 | 4.64* | 0.24 | [4.15, 5.13] |  | 24.92 | 5.74* | 0.23 | [5.28, 6.21] |  | 14.24 | 3.71* | 0.26 | [3.18, 4.23] |  | 15.95 | 3.75* | 0.24 | [3.28, 4.23] |
| [70:30] | 12.03 | 2.83* | 0.24 | [2.36, 3.31] |  | 24.59 | 6.53* | 0.27 | [5.99, 7.06] |  | 17.22 | 5.35* | 0.31 | [4.72, 5.97] |  | 17.77 | 3.53* | 0.20 | [3.13, 3.93] |
| **Hap-sad** |  |  |  |  |  |  |  |  |  |  |  |  |  |  |  |  |  |  |  |
| [30:70] | 24.20 | 6.65* | 0.27 | [6.10, 7.21] |  | 14.88 | 4.10* | 0.28 | [3.54, 4.66] |  | 8.27 | 1.95* | 0.24 | [1.48, 2.43] |  | 8.04 | 1.41* | 0.18 | [1.06, 1.76] |
| [50:50] | 20.86 | 5.05* | 0.24 | [4.56, 5.54] |  | 25.60 | 6.15* | 0.24 | [5.67, 6.64] |  | 15.12 | 4.11* | 0.27 | [3.56, 4.66] |  | 16.99 | 4.16* | 0.24 | [3.66, 4.65] |
| [70:30] | 14.06 | 3.60* | 0.26 | [3.08, 4.11] |  | 28.66 | 7.29* | 0.25 | [6.78, 7.80] |  | 21.74 | 6.11* | 0.28 | [5.54, 6.68] |  | 19.90 | 4.29* | 0.22 | [3.86, 4.73] |
| **Table S1 (Continued)** | | |  |  |  |  |  |  |  |  |  |  |  |  |  |  |  |  |  |
| Fear |  |  |  |  |  |  |  |  |  |  |  |  |  |  |  |  |  |  |  |
|  | Intended | | | | | | | | | | | | | | | | | | |
|  | Ang-fea | | | |  | Disg-fea | | | |  | Fea-hap | | | |  | Fea-sad | | | |
| Non- intended | *t*(42) | *M* ^diff^ | *SE* | CI 95% |  | *t*(42) | *M* ^diff^ | *SE* | CI 95% |  | *t*(42) | *M* ^diff^ | *SE* | CI 95% |  | *t*(42) | *M* ^diff^ | *SE* | CI 95% |
| **Ang-disg** |  |  |  |  |  |  |  |  |  |  |  |  |  |  |  |  |  |  |  |
| [30:70] | 13.91 | 3.19* | 0.23 | [2.73, 3.66] |  | 21.23 | 5.29* | 0.25 | [4.79, 5.79] |  | 7.57 | 2.04* | 0.27 | [1.50, 2.59] |  | 10.78 | 2.60* | 0.24 | [2.11, 3.08] |
| [50:50] | 5.52 | 1.47* | 0.27 | [0.93, 2.00] |  | 10.20 | 1.96* | 0.19 | [1.57, 2.34] |  | 12.47 | 3.46* | 0.28 | [2.90, 4.02] |  | 13.34 | 4.49* | 0.34 | [3.81, 5.17] |
| [70:30] | 6.90 | 1.50* | 0.22 | [1.06, 1.93] |  | 5.17 | 1.02* | 0.20 | [0.62, 1.42] |  | 16.12 | 4.37* | 0.27 | [3.82, 4.92] |  | 22.02 | 5.42* | 0.25 | [4.92, 5.92] |
| **Ang-hap** |  |  |  |  |  |  |  |  |  |  |  |  |  |  |  |  |  |  |  |
| [30:70] | 16.76 | 3.36* | 0.20 | [2.96, 3.77] |  | 27.38 | 5.46* | 0.20 | [5.06, 5.86] |  | 10.24 | 2.21* | 0.22 | [1.78, 2.65] |  | 14.26 | 2.77* | 0.19 | [2.38, 3.16] |
| [50:50] | 9.69 | 2.08* | 0.21 | [1.65, 2.52] |  | 13.07 | 2.57* | 0.20 | [2.18, 2.97] |  | 17.17 | 4.07* | 0.24 | [3.59, 4.55] |  | 18.79 | 5.10* | 0.27 | [4.56, 5.65] |
| [70:30] | 5.00 | 1.21* | 0.24 | [0.72, 1.70] |  | 3.46 | 0.74* | 0.21 | [0.31, 1.17] |  | 15.24 | 4.09* | 0.27 | [3.54, 4.63] |  | 21.78 | 5.13* | 0.24 | [4.66, 5.61] |
| **Ang-sad** |  |  |  |  |  |  |  |  |  |  |  |  |  |  |  |  |  |  |  |
| [30:70] | 14.32 | 3.19* | 0.22 | [2.74, 3.64] |  | 20.14 | 5.29* | 0.26 | [4.76, 5.82] |  | 8.28 | 2.04* | 0.25 | [1.54, 2.54] |  | 11.85 | 2.59* | 0.22 | [2.15, 3.03] |
| [50:50] | 4.97 | 1.11* | 0.22 | [0.66, 1.56] |  | 8.20 | 1.60* | 0.20 | [1.21, 1.99] |  | 12.38 | 3.10* | 0.25 | [2.60, 3.61] |  | 14.87 | 4.13* | 0.28 | [3.57, 4.69] |
| [70:30] | 5.71 | 1.05* | 0.18 | [0.68, 1.43] |  | 2.69 | 0.58 | 0.22 | [0.14, 1.02] |  | 14.24 | 3.93* | 0.28 | [3.37, 4.49] |  | 23.73 | 4.98* | 0.21 | [4.55, 5.40] |
|  |  |  |  |  |  |  |  |  |  |  |  |  |  |  |  |  |  |  |  |
|  |  |  |  |  |  |  |  |  |  |  |  |  |  |  |  |  |  |  |  |
| **Table S1 (Continued)** | | | |  |  |  |  |  |  |  |  |  |  |  |  |  |  |  |  |
| Fear (continue) |  |  |  |  |  |  |  |  |  |  |  |  |  |  |  |  |  |  |  |
|  | Intended | | | | | | | | | | | | | | | | | | |
|  | Ang-fea | | | |  | Disg-fea | | | |  | Fea-hap | | | |  | Fea-sad | | | |
| Non- intended | *t*(42) | *M* ^diff^ | *SE* | CI 95% |  | *t*(42) | *M* ^diff^ | *SE* | CI 95% |  | *t*(42) | *M* ^diff^ | *SE* | CI 95% |  | *t*(42) | *M* ^diff^ | *SE* | CI 95% |
| **Disg-hap** |  |  |  |  |  |  |  |  |  |  |  |  |  |  |  |  |  |  |  |
| [30:70] | 13.89 | 3.16* | 0.23 | [2.70, 3.61] |  | 21.41 | 5.25* | 0.25 | [4.76, 5.75] |  | 9.57 | 2.00* | 0.21 | [1.58, 2.43] |  | 12.84 | 2.56* | 0.20 | [2.16, 2.96] |
| [50:50] | 6.97 | 1.72* | 0.25 | [1.23, 2.22] |  | 9.58 | 2.22* | 0.23 | [1.75, 2.68] |  | 17.26 | 3.72* | 0.22 | [3.28, 4.15] |  | 17.23 | 4.75* | 0.28 | [4.19, 5.3] |
| [70:30] | 1.80 | 0.41 | 0.23 | [-0.05, 0.88] |  | -0.25 | -0.06 | 0.23 | [-0.52, 0.41] |  | 10.77 | 3.29* | 0.31 | [2.67, 3.91] |  | 16.27 | 4.34* | 0.27 | [3.80, 4.88] |
| **Disg-sad** |  |  |  |  |  |  |  |  |  |  |  |  |  |  |  |  |  |  |  |
| [30:70] | 11.62 | 2.45* | 0.21 | [2.03, 2.88] |  | 18.59 | 4.55* | 0.24 | [4.06, 5.04] |  | 5.25 | 1.30* | 0.25 | [0.80, 1.80] |  | 9.45 | 1.86* | 0.20 | [1.46, 2.25] |
| [50:50] | 1.60 | 0.39 | 0.24 | [-0.10, 0.89] |  | 3.74 | 0.88* | 0.24 | [0.41, 1.36] |  | 8.94 | 2.38* | 0.27 | [1.85, 2.92] |  | 11.60 | 3.41* | 0.29 | [2.82, 4.01] |
| [70:30] | -0.97 | -0.22 | 0.22 | [-0.67, 0.23] |  | -3.32 | -0.69* | 0.21 | [-1.11, -0.27] |  | 10.21 | 2.66* | 0.26 | [2.13, 3.18] |  | 15.08 | 3.71* | 0.25 | [3.21, 4.20] |
| **Hap-sad** |  |  |  |  |  |  |  |  |  |  |  |  |  |  |  |  |  |  |  |
| [30:70] | 10.61 | 2.28* | 0.21 | [1.85, 2.71] |  | 18.46 | 4.38* | 0.24 | [3.90, 4.85] |  | 4.83 | 1.13* | 0.23 | [0.66, 1.60] |  | 7.64 | 1.68* | 0.22 | [1.24, 2.13] |
| [50:50] | 7.85 | 1.85* | 0.24 | [1.37, 2.32] |  | 10.87 | 2.34* | 0.22 | [1.91, 2.78] |  | 18.55 | 3.84* | 0.21 | [3.42, 4.26] |  | 18.52 | 4.87* | 0.26 | [4.34, 5.40] |
| [70:30] | 5.21 | 1.33* | 0.25 | [0.81, 1.84] |  | 3.52 | 0.85* | 0.24 | [0.36, 1.34] |  | 15.21 | 4.20* | 0.28 | [3.64, 4.76] |  | 22.52 | 5.25* | 0.23 | [4.78, 5.72] |
|  |  |  |  |  |  |  |  |  |  |  |  |  |  |  |  |  |  |  |  |
|  |  |  |  |  |  |  |  |  |  |  |  |  |  |  |  |  |  |  |  |
| **Table S1 (Continued)** | | | | |  |  |  |  |  |  |  |  |  |  |  |  |  |  |  |
| Happiness |  | | | | | | | | | | | | | | | | | | |
|  | Intended | | | | | | | | | | | | | | | | | | |
|  | Ang-hap | | | |  | Disg-hap | | | |  | Fea-hap | | | |  | Hap-sad | | | |
| Non- intended | *t*(42) | *M* ^diff^ | *SE* | CI 95% |  | *t*(42) | *M* ^diff^ | *SE* | CI 95% |  | *t*(42) | *M* ^diff^ | *SE* | CI 95% |  | *t*(42) | *M* ^diff^ | *SE* | CI 95% |
| **Ang-disg** |  |  |  |  |  |  |  |  |  |  |  |  |  |  |  |  |  |  |  |
| [30:70] | 24.83 | 6.83* | 0.28 | [6.27, 7.38] |  | 26.77 | 7.24* | 0.27 | [6.69, 7.79] |  | 22.03 | 6.83* | 0.31 | [6.20, 7.45] |  | 12.53 | 1.92* | 0.15 | [1.61, 2.23] |
| [50:50] | 17.16 | 5.64* | 0.33 | [4.97, 6.30] |  | 17.48 | 5.79* | 0.33 | [5.12, 6.46] |  | 19.72 | 5.45* | 0.28 | [4.89, 6.01] |  | 19.56 | 5.04* | 0.26 | [4.52, 5.56] |
| [70:30] | 10.32 | 2.74* | 0.27 | [2.21, 3.28] |  | 8.72 | 2.19* | 0.25 | [1.69, 2.70] |  | 11.54 | 2.54* | 0.22 | [2.10, 2.99] |  | 21.01 | 5.43* | 0.26 | [4.91, 5.95] |
| **Ang-fea** |  |  |  |  |  |  |  |  |  |  |  |  |  |  |  |  |  |  |  |
| [30:70] | 24.59 | 7.04* | 0.29 | [6.46, 7.62] |  | 26.89 | 7.45* | 0.28 | [6.89, 8.01] |  | 22.64 | 7.04* | 0.31 | [6.41, 7.67] |  | 13.38 | 2.13* | 0.16 | [1.81, 2.45] |
| [50:50] | 17.53 | 5.65* | 0.32 | [5.00, 6.30] |  | 17.44 | 5.81* | 0.33 | [5.13, 6.48] |  | 19.70 | 5.47* | 0.28 | [4.91, 6.03] |  | 19.49 | 5.05* | 0.26 | [4.53, 5.58] |
| [70:30] | 10.38 | 2.85* | 0.27 | [2.29, 3.40] |  | 9.12 | 2.29* | 0.25 | [1.79, 2.80] |  | 11.90 | 2.64* | 0.22 | [2.20, 3.09] |  | 21.34 | 5.53* | 0.26 | [5.01, 6.05] |
| **Ang-sad** |  |  |  |  |  |  |  |  |  |  |  |  |  |  |  |  |  |  |  |
| [30:70] | 24.68 | 6.91* | 0.28 | [6.34, 7.47] |  | 27.08 | 7.32* | 0.27 | [6.77, 7.86] |  | 23.00 | 6.90* | 0.30 | [6.30, 7.51] |  | 13.34 | 2.00* | 0.15 | [1.69, 2.30] |
| [50:50] | 17.90 | 5.69* | 0.32 | [5.05, 6.33] |  | 18.19 | 5.84* | 0.32 | [5.19, 6.49] |  | 20.51 | 5.50* | 0.27 | [4.96, 6.04] |  | 19.29 | 5.09* | 0.26 | [4.56, 5.62] |
| [70:30] | 10.18 | 2.42* | 0.24 | [1.94, 2.90] |  | 8.65 | 1.87* | 0.22 | [1.43, 2.30] |  | 12.51 | 2.22* | 0.18 | [1.86, 2.57] |  | 20.11 | 5.10* | 0.25 | [4.59, 5.62] |
|  |  |  |  |  |  |  |  |  |  |  |  |  |  |  |  |  |  |  |  |
|  |  |  |  |  |  |  |  |  |  |  |  |  |  |  |  |  |  |  |  |
| **Table S1 (Continued)** | | | | | | | | | | | | | | | | | | | |
| Happiness (continue) |  | | | | | | | | | | | | | | | | | | |
|  | Intended | | | | | | | | | | | | | | | | | | |
|  | Ang-hap | | | |  | Disg-hap | | | |  | Fea-hap | | | |  | Hap-sad | | | |
| Non- intended | *t*(42) | *M* ^diff^ | *SE* | CI 95% |  | *t*(42) | *M* ^diff^ | *SE* | CI 95% |  | *t*(42) | *M* ^diff^ | *SE* | CI 95% |  | *t*(42) | *M* ^diff^ | *SE* | CI 95% |
| **Disg-fea** |  |  |  |  |  |  |  |  |  |  |  |  |  |  |  |  |  |  |  |
| [30:70] | 25.59 | 6.98* | 0.27 | [6.43, 7.53] |  | 26.64 | 7.39* | 0.28 | [6.83, 7.95] |  | 22.79 | 6.98* | 0.31 | [6.36, 7.59] |  | 13.13 | 2.07* | 0.16 | [1.75, 2.39] |
| [50:50] | 17.47 | 5.48* | 0.31 | [4.85, 6.11] |  | 17.88 | 5.64* | 0.32 | [5.00, 6.27] |  | 20.03 | 5.29* | 0.26 | [4.76, 5.83] |  | 19.15 | 4.88* | 0.26 | [4.37, 5.40] |
| [70:30] | 9.85 | 2.78* | 0.28 | [2.21, 3.34] |  | 9.89 | 2.22* | 0.22 | [1.77, 2.68] |  | 11.80 | 2.57* | 0.22 | [2.13, 3.01] |  | 19.81 | 5.46* | 0.28 | [4.90, 6.02] |
| **Disg-sad** |  |  |  |  |  |  |  |  |  |  |  |  |  |  |  |  |  |  |  |
| [30:70] | 25.59 | 7.07* | 0.28 | [6.51, 7.63] |  | 27.15 | 7.48* | 0.28 | [6.92, 8.04] |  | 23.32 | 7.07* | 0.30 | [6.45, 7.68] |  | 14.39 | 2.16* | 0.15 | [1.86, 2.46] |
| [50:50] | 17.49 | 5.69* | 0.33 | [5.04, 6.35] |  | 17.86 | 5.85* | 0.33 | [5.19, 6.51] |  | 19.52 | 5.51* | 0.28 | [4.94, 6.08] |  | 18.84 | 5.10* | 0.27 | [4.55, 5.64] |
| [70:30] | 9.31 | 2.81* | 0.30 | [2.20, 3.42] |  | 8.79 | 2.26* | 0.26 | [1.74, 2.78] |  | 10.78 | 2.61* | 0.24 | [2.12, 3.10] |  | 20.03 | 5.50* | 0.27 | [4.95, 6.05] |
| **Fea-sad** |  |  |  |  |  |  |  |  |  |  |  |  |  |  |  |  |  |  |  |
| [30:70] | 24.74 | 7.08* | 0.29 | [6.50, 7.66] |  | 25.70 | 7.49* | 0.29 | [6.90, 8.08] |  | 22.05 | 7.08* | 0.32 | [6.43, 7.73] |  | 13.42 | 2.17* | 0.16 | [1.84, 2.50] |
| [50:50] | 17.33 | 5.75* | 0.33 | [5.08, 6.42] |  | 17.34 | 5.90* | 0.34 | [5.22, 6.59] |  | 19.66 | 5.56* | 0.28 | [4.99, 6.13] |  | 18.94 | 5.15* | 0.27 | [4.60, 5.70] |
| [70:30] | 9.37 | 2.72* | 0.29 | [2.13, 3.30] |  | 8.83 | 2.17* | 0.25 | [1.67, 2.66] |  | 10.87 | 2.52* | 0.23 | [2.05, 2.98] |  | 20.06 | 5.40* | 0.27 | [4.86, 5.95] |
| **Table S1 (Continued)** | | | |  |  |  |  |  |  |  |  |  |  |  |  |  |  |  |  |
| Sadness |  | | | | | | | | | | | | | | | | | | |
|  | Intended | | | | | | | | | | | | | | | | | | |
|  | Ang-sad | | | |  | Disg-sad | | | |  | Fea-sad | | | |  | Hap-sad | | | |
| Non- intended | *t*(42) | *M* ^diff^ | *SE* | CI 95% |  | *t*(42) | *M* ^diff^ | *SE* | CI 95% |  | *t*(42) | *M* ^diff^ | *SE* | CI 95% |  | *t*(42) | *M* ^diff^ | *SE* | CI 95% |
| **Ang-disg** |  |  |  |  |  |  |  |  |  |  |  |  |  |  |  |  |  |  |  |
| [30:70] | 27.63 | 6.19* | 0.22 | [5.73, 6.64] |  | 26.33 | 6.51* | 0.25 | [6.01, 7.01] |  | 32.72 | 7.08* | 0.22 | [6.64, 7.51] |  | 22.83 | 5.90* | 0.26 | [5.37, 6.42] |
| [50:50] | 17.97 | 3.78* | 0.21 | [3.35, 4.20] |  | 17.22 | 4.30* | 0.25 | [3.79, 4.80] |  | 24.62 | 5.49* | 0.22 | [5.04, 5.94] |  | 16.09 | 4.28* | 0.27 | [3.75, 4.82] |
| [70:30] | 11.32 | 2.25* | 0.20 | [1.85, 2.65] |  | 14.39 | 2.94* | 0.20 | [2.53, 3.35] |  | 13.13 | 3.38* | 0.26 | [2.86, 3.90] |  | 13.72 | 4.09* | 0.30 | [3.49, 4.69] |
| **Ang-fea** |  |  |  |  |  |  |  |  |  |  |  |  |  |  |  |  |  |  |  |
| [30:70] | 23.85 | 4.83* | 0.20 | [4.42, 5.24] |  | 24.17 | 5.15* | 0.21 | [4.72, 5.59] |  | 34.35 | 5.72* | 0.17 | [5.38, 6.06] |  | 21.86 | 4.54* | 0.21 | [4.12, 4.96] |
| [50:50] | 13.55 | 3.14* | 0.23 | [2.67, 3.60] |  | 15.17 | 3.66* | 0.24 | [3.17, 4.15] |  | 21.62 | 4.85* | 0.22 | [4.40, 5.31] |  | 13.14 | 3.64* | 0.28 | [3.08, 4.20] |
| [70:30] | 11.80 | 2.36* | 0.20 | [1.95, 2.76] |  | 14.72 | 3.05* | 0.21 | [2.63, 3.46] |  | 12.46 | 3.48* | 0.28 | [2.92, 4.05] |  | 12.81 | 4.19* | 0.33 | [3.53, 4.85] |
| **Ang-hap** |  |  |  |  |  |  |  |  |  |  |  |  |  |  |  |  |  |  |  |
| [30:70] | 27.04 | 6.64* | 0.25 | [6.14, 7.14] |  | 30.44 | 6.97* | 0.23 | [6.50, 7.43] |  | 36.36 | 7.53* | 0.21 | [7.11, 7.95] |  | 26.47 | 6.35* | 0.24 | [5.86, 6.83] |
| [50:50] | 17.86 | 4.10* | 0.23 | [3.64, 4.57] |  | 18.01 | 4.63* | 0.26 | [4.11, 5.15] |  | 25.01 | 5.82* | 0.23 | [5.35, 6.29] |  | 17.28 | 4.61* | 0.27 | [4.07, 5.15] |
| [70:30] | 12.49 | 2.39* | 0.19 | [2.00, 2.77] |  | 15.04 | 3.08* | 0.20 | [2.66, 3.49] |  | 13.57 | 3.52* | 0.26 | [2.99, 4.04] |  | 14.35 | 4.22* | 0.29 | [3.63, 4.82] |
|  |  |  |  |  |  |  |  |  |  |  |  |  |  |  |  |  |  |  |  |
|  |  |  |  |  |  |  |  |  |  |  |  |  |  |  |  |  |  |  |  |
| **Table S1 (Continued)** | | |  |  |  |  |  |  |  |  |  |  |  |  |  |  |  |  |  |
| Sadness  (continue) |  | | | | | | | | | | | | | | | | | | |
|  | Intended | | | | | | | | | | | | | | | | | | |
|  | Ang-sad | | | |  | Disg-sad | | | |  | Fea-sad | | | |  | Hap-sad | | | |
| Non- intended | *t*(42) | *M* ^diff^ | *SE* | CI 95% |  | *t*(42) | *M* ^diff^ | *SE* | CI 95% |  | *t*(42) | *M* ^diff^ | *SE* | CI 95% |  | *t*(42) | *M* ^diff^ | *SE* | CI 95% |
| **Disg-fea** |  |  |  |  |  |  |  |  |  |  |  |  |  |  |  |  |  |  |  |
| [30:70] | 21.23 | 6.17* | 0.29 | [5.59, 6.76] |  | 27.52 | 6.50* | 0.24 | [6.02, 6.98] |  | 32.87 | 7.07* | 0.21 | [6.63, 7.50] |  | 22.77 | 5.88* | 0.26 | [5.36, 6.41] |
| [50:50] | 12.67 | 2.88* | 0.23 | [2.42, 3.34] |  | 12.60 | 3.41* | 0.27 | [2.86, 3.95] |  | 19.24 | 4.60* | 0.24 | [4.12, 5.08] |  | 12.59 | 3.39* | 0.27 | [2.85, 3.94] |
| [70:30] | 5.67 | 1.43* | 0.25 | [0.92, 1.94] |  | 8.72 | 2.12* | 0.24 | [1.63, 2.61] |  | 9.26 | 2.56* | 0.28 | [2.00, 3.12] |  | 10.62 | 3.27* | 0.31 | [2.65, 3.89] |
| **Disg-hap** |  |  |  |  |  |  |  |  |  |  |  |  |  |  |  |  |  |  |  |
| [30:70] | 26.51 | 6.03* | 0.23 | [5.57, 6.49] |  | 27.55 | 6.35* | 0.23 | [5.89, 6.82] |  | 32.12 | 6.92* | 0.22 | [6.48, 7.35] |  | 24.23 | 5.74* | 0.24 | [5.26, 6.21] |
| [50:50] | 11.78 | 3.38* | 0.29 | [2.80, 3.95] |  | 12.94 | 3.90* | 0.30 | [3.29, 4.51] |  | 18.75 | 5.09* | 0.27 | [4.55, 5.64] |  | 15.25 | 3.88* | 0.25 | [3.37, 4.40] |
| [70:30] | 8.62 | 1.91* | 0.22 | [1.46, 2.36] |  | 11.05 | 2.60* | 0.24 | [2.13, 3.08] |  | 10.55 | 3.04* | 0.29 | [2.46, 3.62] |  | 12.56 | 3.75* | 0.30 | [3.15, 4.35] |
| **Fea-hap** |  |  |  |  |  |  |  |  |  |  |  |  |  |  |  |  |  |  |  |
| [30:70] | 26.81 | 6.40* | 0.24 | [5.91, 6.88] |  | 26.88 | 6.72* | 0.25 | [6.22, 7.23] |  | 34.25 | 7.29* | 0.21 | [6.86, 7.72] |  | 23.92 | 6.10* | 0.26 | [5.59, 6.62] |
| [50:50] | 14.83 | 3.76* | 0.25 | [3.25, 4.27] |  | 17.11 | 4.28* | 0.25 | [3.78, 4.79] |  | 23.81 | 5.48* | 0.23 | [5.01, 5.94] |  | 16.50 | 4.27* | 0.26 | [3.75, 4.79] |
| [70:30] | 6.34 | 1.51* | 0.24 | [1.03, 1.99] |  | 8.82 | 2.20* | 0.25 | [1.69, 2.70] |  | 10.49 | 2.64* | 0.25 | [2.13, 3.14] |  | 11.00 | 3.34* | 0.30 | [2.73, 3.96] |

*Note*. *N* = 43. Abbreviations: ang = anger, disg = disgust, fea = fear, hap = happiness, sad = sadness. 30:70 = The first emotion is intended to be less prominent than the second emotion. 50:50 = Both emotions are intended to be equally prominent. 70:30 = The first emotion is intended to be more prominent than the second emotion. Asterisks indicate significance after correction (Bonferroni corrected alpha level = .0021): * *p* < .0001.

**Table S2**

*Multiple Pairwise Comparisons for 30:70 and 70:30 Proportions Between Rating Scales for Intended Emotion Combinations (Study 1)*

| Emotion Scales  [Emotion Proportion] | *t*(42) | *M* ^diff^ | *SE* | CI 95% |
| --- | --- | --- | --- | --- |
| Anger vs Disgust |  |  |  |  |
| Ang-disg [30:70] | -12.39 | -3.28** | 0.26 | [-3.81, -2.74] |
| Ang-disg [70:30] | 8.77 | 2.55** | 0.33 | [1.97, 3.14] |
| Anger vs Fear |  |  |  |  |
| Ang-fea [30:70] | 3.61 | 1.03* | 0.29 | [0.46, 1.61] |
| Ang-fea [70:30] | 21.44 | 6.36** | 0.31 | [5.76, 6.95] |
| Anger vs Happiness |  |  |  |  |
| Ang-hap [30:70] | -11.95 | -4.16** | 0.35 | [-4.86, -3.46] |
| Ang-hap [70:30] | 10.05 | 3.18** | 0.35 | [2.54, 3.82] |
| Anger vs Sadness |  |  |  |  |
| Ang-sad [30:70] | -5.15 | -1.72** | 0.33 | [-2.39, -1.04] |
| Ang-sad [70:30] | 16.08 | 3.76** | 0.29 | [3.29, 4.24] |
| Disgust vs Fear |  |  |  |  |
| Disg-fea [30:70] | -5.28 | -1.64** | 0.31 | [-2.26, -1.01] |
| Disg-fea [70:30] | 19.14 | 5.74** | 0.35 | [5.13, 6.34] |
| Disgust vs Happiness |  |  |  |  |
| Disg-hap [30:70] | -15.34 | -5.30** | 0.35 | [-6.00, -4.60] |
| Disg-hap [70:30] | 11.86 | 3.83** | 0.33 | [3.18, 4.49] |
| Disgust vs Sadness |  |  |  |  |
| Disg-sad [30:70] | -16.52 | -5.50** | 0.26 | [-6.17, -4.83] |
| Disg-sad [70:30] | 3.23 | 0.91* | 0.31 | [0.34, 1.47] |
|  |  |  |  |  |
| **Table S2 (Continued)** |  |  |  |  |
| Emotion Scales  [Emotion Proportion] | *t*(42) | *M* ^diff^ | *SE* | CI 95% |
| Fear vs Happiness |  |  |  |  |
| Fea-hap [30:70] | -11.53 | -4.35** | 0.29 | [-5.11, -3.59] |
| Fea-hap [70:30] | 7.11 | 2.28** | 0.35 | [1.63, 2.93] |
| Fear vs Sadness |  |  |  |  |
| Fea-sad [30:70] | -15.93 | -4.43** | 0.29 | [-5.00, -3.87] |
| Fea-sad [70:30] | 6.27 | 2.12** | 0.29 | [1.44, 2.81] |
| Happiness vs Sadness |  |  |  |  |
| Hap-sad [30:70] | -15.86 | -4.36** | 0.35 | [-4.92, -3.81] |
| Hap-sad [70:30] | 2.72 | 0.98 | 0.35 | [0.25, 1.70] |

*Note.* *N* = 43. Abbreviations: ang = anger, disg = disgust, fea = fear, hap = happiness, sad = sadness. 30:70 = The first emotion is intended to be less prominent than the second emotion. 50:50 = Both emotions are intended to be equally prominent. 70:30 = The first emotion is intended to be more prominent than the second emotion. Asterisks indicate significance after correction (Bonferroni corrected alpha level = .0025): * *p* < .0025, ** *p* < .00001.

## Table S3

*Multiple Pairwise Comparisons Between Intended and Non-intended Emotion Blends for Respective Emotion Scale (Study 2).*

| Anger |  | | | | | | | | | | | | | | | | | | |
| --- | --- | --- | --- | --- | --- | --- | --- | --- | --- | --- | --- | --- | --- | --- | --- | --- | --- | --- | --- |
|  | Intended | | | | | | | | | | | | | | | | | | |
|  | Ang-disg | | | |  | Ang-fea | | | |  | Ang-hap | | | |  | Ang-sad | | | |
| Non- intended | *t*(38) | *M* ^diff^ | *SE* | CI 95% |  | *t*(38) | *M* ^diff^ | *SE* | CI 95% |  | *t*(38) | *M* ^diff^ | *SE* | CI 95% |  | *t*(38) | *M* ^diff^ | *SE* | CI 95% |
| **Disg-fea** |  |  |  |  |  |  |  |  |  |  |  |  |  |  |  |  |  |  |  |
| [30:70] | 9.45 | 2.26* | 0.24 | [1.77, 2.74] |  | 17.09 | 3.85* | 0.23 | [3.40, 4.31] |  | 8.14 | 1.66* | 0.20 | [1.25, 2.07] |  | 17.52 | 4.16* | 0.24 | [3.68, 4.64] |
| [50:50] | 14.58 | 3.26* | 0.22 | [2.80, 3.71] |  | 21.77 | 5.91* | 0.27 | [5.36, 6.46] |  | 10.92 | 3.05* | 0.28 | [2.49, 3.62] |  | 22.66 | 4.96* | 0.22 | [4.52, 5.40] |
| [70:30] | 17.85 | 3.93* | 0.22 | [3.48, 4.37] |  | 24.49 | 5.76* | 0.24 | [5.29, 6.24] |  | 14.67 | 3.83* | 0.26 | [3.30, 4.36] |  | 17.52 | 4.48* | 0.26 | [3.96, 5.00] |
| **Disg-hap** |  |  |  |  |  |  |  |  |  |  |  |  |  |  |  |  |  |  |  |
| [30:70] | 10.90 | 2.73* | 0.25 | [2.22, 3.23] |  | 16.31 | 4.32* | 0.27 | [3.79, 4.86] |  | 12.12 | 2.13* | 0.18 | [1.77, 2.48] |  | 17.78 | 4.63* | 0.26 | [4.10, 5.16] |
| [50:50] | 17.71 | 4.63* | 0.26 | [4.10, 5.16] |  | 23.86 | 7.28* | 0.30 | [6.66, 7.90] |  | 15.72 | 4.42* | 0.28 | [3.85, 4.99] |  | 24.29 | 6.33* | 0.26 | [5.81, 6.86] |
| [70:30] | 20.81 | 5.50* | 0.26 | [4.96, 6.03] |  | 31.02 | 7.33* | 0.24 | [6.85, 7.81] |  | 23.26 | 5.40* | 0.23 | [4.93, 5.87] |  | 25.22 | 6.05* | 0.24 | [5.57, 6.54] |
| **Disg-sad** |  |  |  |  |  |  |  |  |  |  |  |  |  |  |  |  |  |  |  |
| [30:70] | 10.11 | 2.24* | 0.22 | [1.79, 2.68] |  | 16.22 | 3.83* | 0.24 | [3.35, 4.31] |  | 8.78 | 1.64* | 0.19 | [1.26, 2.01] |  | 17.13 | 4.14* | 0.24 | [3.65, 4.63] |
| [50:50] | 15.16 | 3.98* | 0.26 | [3.45, 4.51] |  | 23.07 | 6.63* | 0.29 | [6.05, 7.21] |  | 12.76 | 3.78* | 0.30 | [3.18, 4.38] |  | 23.13 | 5.69* | 0.25 | [5.19, 6.19] |
| [70:30] | 22.54 | 5.01* | 0.22 | [4.56, 5.46] |  | 25.07 | 6.85* | 0.27 | [6.29, 7.40] |  | 20.65 | 4.91* | 0.24 | [4.43, 5.39] |  | 23.96 | 5.56* | 0.23 | [5.09, 6.03] |
|  |  |  |  |  |  |  |  |  |  |  |  |  |  |  |  |  |  |  |  |
| Anger  (continued) |  | | | | | | | | | | | | | | | | | | |
|  | Intended | | | | | | | | | | | | | | | | | | |
|  | Ang-disg | | | |  | Ang-fea | | | |  | Ang-hap | | | |  | Ang-sad | | | |
| Non- intended | *t*(38) | *M* ^diff^ | *SE* | CI 95% |  | *t*(38) | *M* ^diff^ | *SE* | CI 95% |  | *t*(38) | *M* ^diff^ | *SE* | CI 95% |  | *t*(38) | *M* ^diff^ | *SE* | CI 95% |
| **Fea-hap** |  |  |  |  |  |  |  |  |  |  |  |  |  |  |  |  |  |  |  |
| [30:70] | 11.35 | 2.79* | 0.25 | [2.30, 3.29] |  | 15.76 | 4.39* | 0.28 | [3.83, 4.96] |  | 10.97 | 2.20* | 0.20 | [1.79, 2.60] |  | 16.75 | 4.70* | 0.28 | [4.13, 5.27] |
| [50:50] | 18.38 | 4.73* | 0.26 | [4.21, 5.25] |  | 24.40 | 7.38* | 0.30 | [6.76, 7.99] |  | 15.98 | 4.52* | 0.28 | [3.95, 5.09] |  | 25.82 | 6.43* | 0.25 | [5.93, 6.94] |
| [70:30] | 23.72 | 5.79* | 0.24 | [5.30, 6.29] |  | 33.17 | 7.63* | 0.23 | [7.17, 8.10] |  | 25.93 | 5.70* | 0.22 | [5.25, 6.14] |  | 27.63 | 6.35* | 0.23 | [5.89, 6.82] |
| **Fea-sad** |  |  |  |  |  |  |  |  |  |  |  |  |  |  |  |  |  |  |  |
| [30:70] | 12.88 | 2.76* | 0.21 | [2.32, 3.19] |  | 16.44 | 4.35* | 0.26 | [3.82, 4.89] |  | 10.74 | 2.16* | 0.20 | [1.75, 2.56] |  | 18.91 | 4.66* | 0.25 | [4.16, 5.16] |
| [50:50] | 17.49 | 4.59* | 0.26 | [4.06, 5.13] |  | 24.19 | 7.24* | 0.30 | [6.64, 7.85] |  | 15.18 | 4.39* | 0.29 | [3.80, 4.97] |  | 24.26 | 6.30* | 0.26 | [5.77, 6.82] |
| [70:30] | 22.99 | 5.54* | 0.24 | [5.05, 6.03] |  | 31.14 | 7.38* | 0.24 | [6.90, 7.86] |  | 23.18 | 5.44* | 0.23 | [4.96, 5.92] |  | 26.87 | 6.09* | 0.23 | [5.63, 6.55] |
| **Hap-sad** |  |  |  |  |  |  |  |  |  |  |  |  |  |  |  |  |  |  |  |
| [30:70] | 13.19 | 2.93* | 0.22 | [2.48, 3.38] |  | 17.43 | 4.53* | 0.26 | [4.00, 5.06] |  | 11.16 | 2.33* | 0.21 | [1.91, 2.76] |  | 18.78 | 4.84* | 0.26 | [4.32, 5.36] |
| [50:50] | 17.19 | 4.45* | 0.26 | [3.93, 4.98] |  | 22.40 | 7.10* | 0.32 | [6.46, 7.74] |  | 15.17 | 4.25* | 0.28 | [3.68, 4.81] |  | 23.16 | 6.16* | 0.27 | [5.62, 6.70] |
| [70:30] | 22.59 | 5.74* | 0.25 | [5.22, 6.25] |  | 32.16 | 7.58* | 0.24 | [7.10, 8.05] |  | 24.53 | 5.64* | 0.23 | [5.18, 6.11] |  | 26.66 | 6.29* | 0.24 | [5.82, 6.77] |

|  |  |  |  |  |  |  |  |  |  |  |  |  |  |  |  |  |  |  |  |
| --- | --- | --- | --- | --- | --- | --- | --- | --- | --- | --- | --- | --- | --- | --- | --- | --- | --- | --- | --- |
| Disgust |  |  |  |  |  |  |  |  |  |  |  |  |  |  |  |  |  |  |  |
|  | Intended | | | | | | | | | | | | | | | | | | |
|  | Ang-disg | | | |  | Disg-fea | | | |  | Disg-hap | | | |  | Disg-sad | | | |
| Non- intended | *t*(38) | *M* ^diff^ | *SE* | CI 95% |  | *t*(38) | *M* ^diff^ | *SE* | CI 95% |  | *t*(38) | *M* ^diff^ | *SE* | CI 95% |  | *t*(38) | *M* ^diff^ | *SE* | CI 95% |
| **Ang-fea** |  |  |  |  |  |  |  |  |  |  |  |  |  |  |  |  |  |  |  |
| [30:70] | 22.44 | 6.16* | 0.27 | [5.61, 6.72] |  | 14.68 | 3.30* | 0.22 | [2.84, 3.75] |  | 7.15 | 1.66* | 0.23 | [1.19, 2.13] |  | 4.86 | 0.82* | 0.17 | [0.48, 1.16] |
| [50:50] | 19.92 | 4.54* | 0.23 | [4.08, 5.00] |  | 20.61 | 5.71* | 0.28 | [5.15, 6.27] |  | 15.52 | 4.36* | 0.28 | [3.79, 4.93] |  | 12.84 | 4.25* | 0.33 | [3.58, 4.92] |
| [70:30] | 13.85 | 2.43* | 0.18 | [2.07, 2.78] |  | 24.73 | 6.63* | 0.27 | [6.09, 7.18] |  | 21.11 | 5.78* | 0.27 | [5.22, 6.33] |  | 16.37 | 2.99* | 0.18 | [2.62, 3.36] |
| **Ang-hap** |  |  |  |  |  |  |  |  |  |  |  |  |  |  |  |  |  |  |  |
| [30:70] | 25.92 | 6.48* | 0.25 | [5.97, 6.98] |  | 15.72 | 3.62* | 0.23 | [3.15, 4.08] |  | 9.90 | 1.97* | 0.20 | [1.57, 2.38] |  | 6.03 | 1.14* | 0.19 | [0.75, 1.52] |
| [50:50] | 17.89 | 4.49* | 0.25 | [3.98, 4.99] |  | 19.34 | 5.66* | 0.29 | [5.07, 6.25] |  | 15.36 | 4.30* | 0.28 | [3.74, 4.87] |  | 12.42 | 4.20* | 0.34 | [3.51, 4.88] |
| [70:30] | 15.73 | 2.59* | 0.16 | [2.26, 2.92] |  | 22.55 | 6.79* | 0.30 | [6.18, 7.40] |  | 20.96 | 5.94* | 0.28 | [5.37, 6.51] |  | 15.88 | 3.15* | 0.20 | [2.75, 3.55] |
| **Ang-sad** |  |  |  |  |  |  |  |  |  |  |  |  |  |  |  |  |  |  |  |
| [30:70] | 23.76 | 6.39* | 0.27 | [5.85, 6.94] |  | 16.35 | 3.53* | 0.22 | [3.09, 3.97] |  | 8.52 | 1.89* | 0.22 | [1.44, 2.34] |  | 6.58 | 1.05* | 0.16 | [0.73, 1.37] |
| [50:50] | 17.03 | 4.33* | 0.25 | [3.81, 4.84] |  | 17.33 | 5.50* | 0.32 | [4.86, 6.14] |  | 13.33 | 4.15* | 0.31 | [3.52, 4.77] |  | 11.72 | 4.04* | 0.34 | [3.34, 4.74] |
| [70:30] | 13.26 | 2.53* | 0.19 | [2.14, 2.91] |  | 22.29 | 6.73* | 0.30 | [6.12, 7.34] |  | 18.08 | 5.88* | 0.32 | [5.22, 6.53] |  | 15.84 | 3.09* | 0.19 | [2.69, 3.48] |
|  |  |  |  |  |  |  |  |  |  |  |  |  |  |  |  |  |  |  |  |
| Disgust  (continued) |  |  |  |  |  |  |  |  |  |  |  |  |  |  |  |  |  |  |  |
|  | Intended | | | | | | | | | | | | | | | | | | |
|  | Ang-disg | | | |  | Disg-fea | | | |  | Disg-hap | | | |  | Disg-sad | | | |
| Non- intended | *t*(38) | *M* ^diff^ | *SE* | CI 95% |  | *t*(38) | *M* ^diff^ | *SE* | CI 95% |  | *t*(38) | *M* ^diff^ | *SE* | CI 95% |  | *t*(38) | *M* ^diff^ | *SE* | CI 95% |
| **Fea-hap** |  |  |  |  |  |  |  |  |  |  |  |  |  |  |  |  |  |  |  |
| [30:70] | 24.43 | 6.38* | 0.26 | [5.85, 6.90] |  | 14.30 | 3.51* | 0.25 | [3.02, 4.01] |  | 9.22 | 1.87* | 0.20 | [1.46, 2.28] |  | 5.19 | 1.03* | 0.20 | [0.63, 1.44] |
| [50:50] | 16.75 | 4.33* | 0.26 | [3.81, 4.85] |  | 19.63 | 5.50* | 0.28 | [4.93, 6.07] |  | 16.34 | 4.15* | 0.25 | [3.63, 4.66] |  | 13.25 | 4.04* | 0.30 | [3.42, 4.66] |
| [70:30] | 11.27 | 2.30* | 0.20 | [1.89, 2.71] |  | 20.84 | 6.50* | 0.31 | [5.87, 7.14] |  | 18.67 | 5.65* | 0.30 | [5.04, 6.26] |  | 15.43 | 2.86* | 0.19 | [2.48, 3.23] |
| **Fea-sad** |  |  |  |  |  |  |  |  |  |  |  |  |  |  |  |  |  |  |  |
| [30:70] | 24.80 | 6.66* | 0.27 | [6.12, 7.21] |  | 16.66 | 3.80* | 0.23 | [3.34, 4.26] |  | 10.79 | 2.16* | 0.20 | [1.75, 2.56] |  | 7.70 | 1.32* | 0.17 | [0.97, 1.67] |
| [50:50] | 17.03 | 4.34* | 0.26 | [3.83, 4.86] |  | 18.99 | 5.51* | 0.29 | [4.93, 6.10] |  | 14.20 | 4.16* | 0.29 | [3.57, 4.75] |  | 12.44 | 4.05* | 0.33 | [3.39, 4.71] |
| [70:30] | 11.95 | 2.60* | 0.22 | [2.16, 3.04] |  | 24.22 | 6.80* | 0.28 | [6.23, 7.37] |  | 19.19 | 5.95* | 0.31 | [5.32, 6.58] |  | 18.31 | 3.16* | 0.17 | [2.81, 3.51] |
| **Hap-sad** |  |  |  |  |  |  |  |  |  |  |  |  |  |  |  |  |  |  |  |
| [30:70] | 23.19 | 6.28* | 0.27 | [5.73, 6.83] |  | 14.16 | 3.42* | 0.24 | [2.93, 3.91] |  | 7.92 | 1.78* | 0.22 | [1.32, 2.23] |  | 5.07 | 0.94* | 0.19 | [0.57, 1.32] |
| [50:50] | 18.68 | 4.68* | 0.25 | [4.17, 5.18] |  | 19.07 | 5.85* | 0.31 | [5.23, 6.47] |  | 15.11 | 4.49* | 0.30 | [3.89, 5.09] |  | 13.10 | 4.38* | 0.33 | [3.71, 5.06] |
| [70:30] | 16.35 | 3.04* | 0.19 | [2.67, 3.42] |  | 24.92 | 7.25* | 0.29 | [6.66, 7.84] |  | 22.16 | 6.39* | 0.29 | [5.81, 6.98] |  | 21.94 | 3.60* | 0.16 | [3.27, 3.94] |

|  |  |  |  |  |  |  |  |  |  |  |  |  |  |  |  |  |  |  |  |
| --- | --- | --- | --- | --- | --- | --- | --- | --- | --- | --- | --- | --- | --- | --- | --- | --- | --- | --- | --- |
| Fear |  |  |  |  |  |  |  |  |  |  |  |  |  |  |  |  |  |  |  |
|  | Intended | | | | | | | | | | | | | | | | | | |
|  | Ang-fea | | | |  | Disg-fea | | | |  | Fea-hap | | | |  | Fea-sad | | | |
| Non- intended | *t*(38) | *M* ^diff^ | *SE* | CI 95% |  | *t*(38) | *M* ^diff^ | *SE* | CI 95% |  | *t*(38) | *M* ^diff^ | *SE* | CI 95% |  | *t*(38) | *M* ^diff^ | *SE* | CI 95% |
| **Ang-disg** |  |  |  |  |  |  |  |  |  |  |  |  |  |  |  |  |  |  |  |
| [30:70] | 11.28 | 2.90* | 0.26 | [2.38, 3.42] |  | 23.29 | 5.30* | 0.23 | [4.84, 5.76] |  | 6.46 | 1.38* | 0.21 | [0.94, 1.81] |  | 9.68 | 1.70* | 0.18 | [1.34, 2.05] |
| [50:50] | 6.85 | 1.44* | 0.21 | [1.01, 1.87] |  | 10.32 | 2.23* | 0.22 | [1.79, 2.66] |  | 11.90 | 3.12* | 0.26 | [2.59, 3.65] |  | 15.03 | 4.43* | 0.29 | [3.83, 5.02] |
| [70:30] | 6.07 | 1.43* | 0.24 | [0.95, 1.91] |  | 2.53 | 0.45 | 0.18 | [0.09, 0.82] |  | 17.98 | 4.69* | 0.26 | [4.16, 5.22] |  | 19.80 | 4.98* | 0.25 | [4.47, 5.49] |
| **Ang-hap** |  |  |  |  |  |  |  |  |  |  |  |  |  |  |  |  |  |  |  |
| [30:70] | 13.17 | 3.31* | 0.25 | [2.80, 3.82] |  | 28.24 | 5.71* | 0.20 | [5.30, 6.12] |  | 9.02 | 1.79* | 0.20 | [1.39, 2.19] |  | 10.77 | 2.11* | 0.20 | [1.71, 2.51] |
| [50:50] | 8.01 | 1.89* | 0.24 | [1.41, 2.37] |  | 12.00 | 2.68* | 0.22 | [2.23, 3.13] |  | 14.09 | 3.57* | 0.25 | [3.06, 4.09] |  | 16.08 | 4.88* | 0.30 | [4.27, 5.49] |
| [70:30] | 4.80 | 1.05* | 0.22 | [0.61, 1.49] |  | 0.42 | 0.07 | 0.16 | [-0.26, 0.40] |  | 17.67 | 4.30* | 0.24 | [3.81, 4.80] |  | 20.89 | 4.60* | 0.22 | [4.15, 5.04] |
| **Ang-sad** |  |  |  |  |  |  |  |  |  |  |  |  |  |  |  |  |  |  |  |
| [30:70] | 10.63 | 2.54* | 0.24 | [2.06, 3.03] |  | 21.27 | 4.94* | 0.23 | [4.47, 5.42] |  | 4.32 | 1.02* | 0.24 | [0.54, 1.50] |  | 7.20 | 1.34* | 0.19 | [0.96, 1.72] |
| [50:50] | 4.98 | 0.92* | 0.19 | [0.55, 1.30] |  | 7.45 | 1.71* | 0.23 | [1.25, 2.17] |  | 10.45 | 2.60* | 0.25 | [2.10, 3.11] |  | 13.56 | 3.91* | 0.29 | [3.33, 4.49] |
| [70:30] | 3.61 | 0.86* | 0.24 | [0.38, 1.34] |  | -0.63 | -0.12 | 0.19 | [-0.50, 0.26] |  | 16.40 | 4.12* | 0.25 | [3.61, 4.62] |  | 17.16 | 4.41* | 0.26 | [3.89, 4.93] |
|  |  |  |  |  |  |  |  |  |  |  |  |  |  |  |  |  |  |  |  |
|  |  |  |  |  |  |  |  |  |  |  |  |  |  |  |  |  |  |  |  |
|  |  |  |  |  |  |  |  |  |  |  |  |  |  |  |  |  |  |  |  |
| Fear  (continued) |  |  |  |  |  |  |  |  |  |  |  |  |  |  |  |  |  |  |  |
|  | Intended | | | | | | | | | | | | | | | | | | |
|  | Ang-fea | | | |  | Disg-fea | | | |  | Fea-hap | | | |  | Fea-sad | | | |
| Non- intended | *t*(38) | *M* ^diff^ | *SE* | CI 95% |  | *t*(38) | *M* ^diff^ | *SE* | CI 95% |  | *t*(38) | *M* ^diff^ | *SE* | CI 95% |  | *t*(38) | *M* ^diff^ | *SE* | CI 95% |
| **Disg-hap** |  |  |  |  |  |  |  |  |  |  |  |  |  |  |  |  |  |  |  |
| [30:70] | 12.97 | 3.23* | 0.25 | [2.73, 3.74] |  | 27.92 | 5.63* | 0.20 | [5.22, 6.04] |  | 9.32 | 1.71* | 0.18 | [1.34, 2.08] |  | 10.73 | 2.03* | 0.19 | [1.65, 2.41] |
| [50:50] | 6.18 | 1.53* | 0.25 | [1.03, 2.03] |  | 11.65 | 2.31* | 0.20 | [1.91, 2.71] |  | 13.05 | 3.21* | 0.25 | [2.71, 3.70] |  | 15.39 | 4.51* | 0.29 | [3.92, 5.11] |
| [70:30] | -0.48 | -0.12 | 0.25 | [-0.62, 0.38] |  | -5.00 | -1.10* | 0.22 | [-1.54, -0.65] |  | 11.02 | 3.14* | 0.28 | [2.56, 3.71] |  | 12.57 | 3.43* | 0.27 | [2.88, 3.98] |
| **Disg-sad** |  |  |  |  |  |  |  |  |  |  |  |  |  |  |  |  |  |  |  |
| [30:70] | 10.32 | 2.71* | 0.26 | [2.18, 3.25] |  | 19.51 | 5.12* | 0.26 | [4.58, 5.65] |  | 5.20 | 1.19* | 0.23 | [0.73, 1.66] |  | 9.06 | 1.51* | 0.17 | [1.17, 1.85] |
| [50:50] | 1.22 | 0.28 | 0.23 | [-0.18, 0.74] |  | 5.45 | 1.06* | 0.20 | [0.67, 1.46] |  | 7.33 | 1.96* | 0.27 | [1.42, 2.50] |  | 12.33 | 3.26* | 0.26 | [2.73, 3.80] |
| [70:30] | -1.62 | -0.39 | 0.24 | [-0.88, 0.10] |  | -7.58 | -1.37* | 0.18 | [-1.74, -1.01] |  | 11.45 | 2.86* | 0.25 | [2.36, 3.37] |  | 12.74 | 3.16* | 0.25 | [2.66, 3.66] |
| **Hap-sad** |  |  |  |  |  |  |  |  |  |  |  |  |  |  |  |  |  |  |  |
| [30:70] | 11.06 | 2.82* | 0.25 | [2.30, 3.33] |  | 25.42 | 5.22* | 0.21 | [4.80, 5.63] |  | 6.52 | 1.29* | 0.20 | [0.89, 1.70] |  | 7.21 | 1.62* | 0.22 | [1.16, 2.07] |
| [50:50] | 8.26 | 1.88* | 0.23 | [1.42, 2.34] |  | 13.03 | 2.67* | 0.20 | [2.25, 3.08] |  | 13.97 | 3.56* | 0.25 | [3.04, 4.08] |  | 16.89 | 4.87* | 0.29 | [4.28, 5.45] |
| [70:30] | 5.21 | 1.25* | 0.24 | [0.76, 1.73] |  | 1.58 | 0.27 | 0.17 | [-0.08, 0.61] |  | 18.34 | 4.50* | 0.25 | [4.01, 5.00] |  | 21.17 | 4.80* | 0.23 | [4.34, 5.26] |

|  |  | |  | |  | |  | |  |  |  |  | |  | |  |  | |  |  |  |  | |  | |  | |  | |  |
| --- | --- | --- | --- | --- | --- | --- | --- | --- | --- | --- | --- | --- | --- | --- | --- | --- | --- | --- | --- | --- | --- | --- | --- | --- | --- | --- | --- | --- | --- | --- |
| Happiness |  | | | | | | | | | | | | | | | | | | | | | | | | | | | | | |
|  | Intended | | | | | | | | | | | | | | | | | | | | | | | | | | | | | |
|  | Ang-hap | | | | | | | |  | Disg-hap | | | | | |  | Fea-hap | | | | |  | | Hap-sad | | | | | | |
| Non- intended | *t*(38) | | *M* ^diff^ | | *SE* | | CI 95% | |  | *t*(38) | *M* ^diff^ | *SE* | | CI 95% | |  | *t*(38) | | *M* ^diff^ | *SE* | CI 95% |  | | *t*(38) | | *M* ^diff^ | | *SE* | | CI 95% |
| **Ang-disg** |  | |  | |  | |  | |  |  |  |  | |  | |  |  | |  |  |  |  | |  | |  | |  | |  |
| [30:70] | 22.38 | | 6.94* | | 0.31 | | [6.32, 7.57] | |  | 25.70 | 7.19* | 0.28 | | [6.63, 7.76] | |  | 22.35 | | 6.65* | 0.30 | [6.05, 7.25] |  | | 11.02 | | 1.90* | | 0.17 | | [1.55, 2.25] |
| [50:50] | 18.04 | | 5.30* | | 0.29 | | [4.71, 5.90] | |  | 15.54 | 6.07* | 0.39 | | [5.28, 6.86] | |  | 22.61 | | 5.52* | 0.24 | [5.02, 6.01] |  | | 18.40 | | 5.15* | | 0.28 | | [4.58, 5.71] |
| [70:30] | 11.03 | | 2.87* | | 0.26 | | [2.34, 3.39] | |  | 9.43 | 2.96* | 0.31 | | [2.32, 3.59] | |  | 12.04 | | 2.82* | 0.23 | [2.35, 3.29] |  | | 18.97 | | 5.38* | | 0.28 | | [4.80, 5.95] |
| **Ang-fea** |  | |  | |  | |  | |  |  |  |  | |  | |  |  | |  |  |  |  | |  | |  | |  | |  |
| [30:70] | 23.01 | | 7.09* | | 0.31 | | [6.47, 7.72] | |  | 25.27 | 7.34* | 0.29 | | [6.75, 7.93] | |  | 22.04 | | 6.80* | 0.31 | [6.17, 7.42] |  | | 12.53 | | 2.05* | | 0.16 | | [1.72, 2.38] |
| [50:50] | 17.89 | | 5.29* | | 0.30 | | [4.70, 5.89] | |  | 15.36 | 6.06* | 0.39 | | [5.26, 6.86] | |  | 22.10 | | 5.51* | 0.25 | [5.00, 6.01] |  | | 18.02 | | 5.14* | | 0.28 | | [4.56, 5.71] |
| [70:30] | 10.96 | | 2.88* | | 0.26 | | [2.35, 3.41] | |  | 9.39 | 2.97* | 0.32 | | [2.33, 3.61] | |  | 11.94 | | 2.83* | 0.24 | [2.35, 3.31] |  | | 18.75 | | 5.39* | | 0.29 | | [4.81, 5.97] |
| **Ang-sad** |  | |  | |  | |  | |  |  |  |  | |  | |  |  | |  |  |  |  | |  | |  | |  | |  |
| [30:70] | 22.77 | | 6.94* | | 0.30 | | [6.33, 7.56] | |  | 24.87 | 7.19* | 0.29 | | [6.61, 7.78] | |  | 21.90 | | 6.65* | 0.30 | [6.03, 7.26] |  | | 10.90 | | 1.90* | | 0.17 | | [1.55, 2.25] |
| [50:50] | 18.54 | | 4.97* | | 0.27 | | [4.43, 5.52] | |  | 15.23 | 5.74* | 0.38 | | [4.98, 6.51] | |  | 22.10 | | 5.19* | 0.23 | [4.71, 5.66] |  | | 17.08 | | 4.82* | | 0.28 | | [4.25, 5.39] |
| [70:30] | 9.87 | | 2.41* | | 0.24 | | [1.91, 2.90] | |  | 8.79 | 2.50* | 0.28 | | [1.92, 3.07] | |  | 10.93 | | 2.36* | 0.22 | [1.92, 2.80] |  | | 17.96 | | 4.91* | | 0.27 | | [4.36, 5.47] |
|  |  | |  | |  | |  | |  |  |  |  | |  | |  |  | |  |  |  |  | |  | |  | |  | |  |
|  |  | |  | |  | |  | |  |  |  |  | |  | |  |  | |  |  |  |  | |  | |  | |  | |  |
|  |  | | | | | | | | | | | | | | | | | | | | | | | | | | | | | |
| Happiness (continued) |  | | | | | | | | | | | | | | | | | | | | | | | | | | | | | |
|  | Intended | | | | | | | | | | | | | | | | | | | | | | | | | | | | | |
|  | Ang-hap | | | | | | | |  | Disg-hap | | | | | |  | Fea-hap | | | | |  | | Hap-sad | | | | | | |
| Non- intended | *t*(38) | | *M* ^diff^ | | *SE* | | CI 95% | |  | *t*(38) | *M* ^diff^ | *SE* | | CI 95% | |  | *t*(38) | | *M* ^diff^ | *SE* | CI 95% |  | | *t*(38) | | *M* ^diff^ | | *SE* | | CI 95% |
| **Disg-fea** |  | |  | |  | |  | |  |  |  |  | |  | |  |  | |  |  |  |  | |  | |  | |  | |  |
| [30:70] | 22.61 | | 7.11* | | 0.31 | | [6.47, 7.74] | |  | 24.79 | 7.35* | 0.30 | | [6.75, 7.96] | |  | 21.77 | | 6.81* | 0.31 | [6.18, 7.45] |  | | 12.36 | | 2.06* | | 0.17 | | [1.72, 2.40] |
| [50:50] | 17.64 | | 5.12* | | 0.29 | | [4.53, 5.70] | |  | 15.11 | 5.88* | 0.39 | | [5.10, 6.67] | |  | 21.22 | | 5.33* | 0.25 | [4.82, 5.84] |  | | 17.30 | | 4.96* | | 0.29 | | [4.38, 5.54] |
| [70:30] | 11.20 | | 2.71* | | 0.24 | | [2.22, 3.20] | |  | 9.40 | 2.80* | 0.30 | | [2.20, 3.41] | |  | 11.97 | | 2.67* | 0.22 | [2.22, 3.12] |  | | 18.55 | | 5.22* | | 0.28 | | [4.65, 5.79] |
| **Disg-sad** |  | |  | |  | |  | |  |  |  |  | |  | |  |  | |  |  |  |  | |  | |  | |  | |  |
| [30:70] | 22.89 | | 7.06* | | 0.31 | | [6.44, 7.69] | |  | 25.18 | 7.31* | 0.29 | | [6.72, 7.90] | |  | 21.98 | | 6.77* | 0.31 | [6.15, 7.39] |  | | 12.31 | | 2.02* | | 0.16 | | [1.69, 2.35] |
| [50:50] | 17.01 | | 5.21* | | 0.31 | | [4.59, 5.82] | |  | 15.12 | 5.97* | 0.40 | | [5.17, 6.77] | |  | 21.60 | | 5.42* | 0.25 | [4.91, 5.93] |  | | 17.18 | | 5.05* | | 0.29 | | [4.45, 5.64] |
| [70:30] | 10.58 | | 2.81* | | 0.27 | | [2.27, 3.35] | |  | 9.23 | 2.90* | 0.31 | | [2.27, 3.54] | |  | 11.45 | | 2.76* | 0.24 | [2.28, 3.25] |  | | 18.66 | | 5.32* | | 0.29 | | [4.74, 5.90] |
| **Fea-sad** | |  | |  | |  | |  |  |  |  | |  | |  |  |  |  | |  |  | |  |  |  | |  | |  | |
| [30:70] | | 22.62 | | 7.15* | | 0.32 | | [6.51, 7.79] |  | 24.92 | 7.40* | | 0.30 | | [6.80, 8.00] |  | 21.80 | 6.86* | | 0.31 | [6.22, 7.50] | |  | 12.44 | 2.11* | | 0.17 | | [1.76, 2.45] | |
| [50:50] | | 17.81 | | 5.32* | | 0.30 | | [4.72, 5.93] |  | 15.43 | 6.09* | | 0.39 | | [5.29, 6.89] |  | 22.56 | 5.54* | | 0.25 | [5.04, 6.04] | |  | 18.15 | 5.17* | | 0.28 | | [4.59, 5.74] | |
| [70:30] | | 10.39 | | 2.69* | | 0.26 | | [2.16, 3.21] |  | 8.98 | 2.78* | | 0.31 | | [2.15, 3.40] |  | 11.51 | 2.64* | | 0.23 | [2.18, 3.11] | |  | 18.69 | 5.20* | | 0.28 | | [4.63, 5.76] | |

|  |  | |  | |  | |  | |  |  |  |  |  |  |  | |  |  |  |  | |  | |  | |  | |  |
| --- | --- | --- | --- | --- | --- | --- | --- | --- | --- | --- | --- | --- | --- | --- | --- | --- | --- | --- | --- | --- | --- | --- | --- | --- | --- | --- | --- | --- |
| Sadness |  | | | | | | | | | | | | | | | | | | | | | | | | | | | |
|  | Intended | | | | | | | | | | | | | | | | | | | | | | | | | | | |
|  | Ang-sad | | | | | | | |  | Disg-sad | | | |  | Fea-sad | | | | |  | | Hap-sad | | | | | | |
| Non- intended | *t*(38) | | *M* ^diff^ | | *SE* | | CI 95% | |  | *t*(38) | *M* ^diff^ | *SE* | CI 95% |  | *t*(38) | | *M* ^diff^ | *SE* | CI 95% |  | | *t*(38) | | *M* ^diff^ | | *SE* | | CI 95% |
| **Ang-disg** |  | |  | |  | |  | |  |  |  |  |  |  |  | |  |  |  |  | |  | |  | |  | |  |
| [30:70] | 20.96 | | 6.08* | | 0.29 | | [5.49, 6.66] | |  | 26.99 | 6.91* | 0.26 | [6.39, 7.43] |  | 27.88 | | 7.47* | 0.27 | [6.93, 8.02] |  | | 19.77 | | 6.00* | | 0.30 | | [5.39, 6.62] |
| [50:50] | 13.48 | | 3.81* | | 0.28 | | [3.24, 4.38] | |  | 16.94 | 4.24* | 0.25 | [3.74, 4.75] |  | 16.79 | | 5.10* | 0.30 | [4.48, 5.71] |  | | 17.18 | | 4.24* | | 0.25 | | [3.74, 4.74] |
| [70:30] | 8.65 | | 1.94* | | 0.22 | | [1.48, 2.39] | |  | 16.69 | 3.12* | 0.19 | [2.74, 3.50] |  | 17.07 | | 3.19* | 0.19 | [2.81, 3.57] |  | | 14.66 | | 3.79* | | 0.26 | | [3.27, 4.31] |
| **Ang-fea** |  | |  | |  | |  | |  |  |  |  |  |  |  | |  |  |  |  | |  | |  | |  | |  |
| [30:70] | 19.06 | | 4.64* | | 0.24 | | [4.14, 5.13] | |  | 24.67 | 5.47* | 0.22 | [5.02, 5.92] |  | 24.79 | | 6.03* | 0.24 | [5.54, 6.53] |  | | 16.28 | | 4.56* | | 0.28 | | [4.00, 5.13] |
| [50:50] | 15.18 | | 3.50* | | 0.23 | | [3.03, 3.97] | |  | 15.31 | 3.94* | 0.26 | [3.42, 4.46] |  | 15.05 | | 4.79* | 0.32 | [4.15, 5.43] |  | | 14.51 | | 3.93* | | 0.27 | | [3.38, 4.48] |
| [70:30] | 11.34 | | 2.19* | | 0.19 | | [1.80, 2.58] | |  | 17.54 | 3.38* | 0.19 | [2.99, 3.77] |  | 16.73 | | 3.45* | 0.21 | [3.03, 3.87] |  | | 15.15 | | 4.05* | | 0.27 | | [3.51, 4.59] |
| **Ang-hap** |  | |  | |  | |  | |  |  |  |  |  |  |  | |  |  |  |  | |  | |  | |  | |  |
| [30:70] | 23.65 | | 6.41* | | 0.27 | | [5.86, 6.96] | |  | 30.04 | 7.24* | 0.24 | [6.76, 7.73] |  | 31.66 | | 7.81* | 0.25 | [7.31, 8.31] |  | | 21.36 | | 6.34* | | 0.30 | | [5.74, 6.94] |
| [50:50] | 14.06 | | 4.05* | | 0.29 | | [3.46, 4.63] | |  | 16.04 | 4.48* | 0.28 | [3.92, 5.05] |  | 16.89 | | 5.34* | 0.32 | [4.70, 5.98] |  | | 18.22 | | 4.48* | | 0.25 | | [3.98, 4.98] |
| [70:30] | 10.22 | | 2.14* | | 0.21 | | [1.72, 2.57] | |  | 19.87 | 3.32* | 0.17 | [2.99, 3.66] |  | 17.75 | | 3.40* | 0.19 | [3.01, 3.78] |  | | 15.48 | | 4.00* | | 0.26 | | [3.47, 4.52] |
|  |  | |  | |  | |  | |  |  |  |  |  |  |  | |  |  |  |  | |  | |  | |  | |  |
|  |  | |  | |  | |  | |  |  |  |  |  |  |  | |  |  |  |  | |  | |  | |  | |  |
| Sadness  (continued) |  | | | | | | | | | | | | | | | | | | | | | | | | | | | |
|  | Intended | | | | | | | | | | | | | | | | | | | | | | | | | | | |
|  | Ang-sad | | | | | | | |  | Disg-sad | | | |  | Fea-sad | | | | |  | | Hap-sad | | | | | | |
| Non- intended | *t*(38) | | *M* ^diff^ | | *SE* | | CI 95% | |  | *t*(38) | *M* ^diff^ | *SE* | CI 95% |  | *t*(38) | | *M* ^diff^ | *SE* | CI 95% |  | | *t*(38) | | *M* ^diff^ | | *SE* | | CI 95% |
| **Disg-fea** |  | |  | |  | |  | |  |  |  |  |  |  |  | |  |  |  |  | |  | |  | |  | |  |
| [30:70] | 20.80 | | 5.86* | | 0.28 | | [5.29, 6.43] | |  | 27.30 | 6.69* | 0.25 | [6.20, 7.19] |  | 27.75 | | 7.26* | 0.26 | [6.73, 7.79] |  | | 19.34 | | 5.79* | | 0.30 | | [5.18, 6.39] |
| [50:50] | 11.87 | | 2.86* | | 0.24 | | [2.37, 3.35] | |  | 13.23 | 3.29* | 0.25 | [2.79, 3.80] |  | 15.42 | | 4.15* | 0.27 | [3.60, 4.69] |  | | 12.80 | | 3.29* | | 0.26 | | [2.77, 3.81] |
| [70:30] | 7.83 | | 1.72* | | 0.22 | | [1.27, 2.16] | |  | 16.04 | 2.90* | 0.18 | [2.54, 3.27] |  | 15.98 | | 2.97* | 0.19 | [2.60, 3.35] |  | | 14.36 | | 3.57* | | 0.25 | | [3.07, 4.08] |
| **Disg-hap** |  | |  | |  | |  | |  |  |  |  |  |  |  | |  |  |  |  | |  | |  | |  | |  |
| [30:70] | 21.54 | | 5.94* | | 0.28 | | [5.38, 6.49] | |  | 27.95 | 6.77* | 0.24 | [6.28, 7.26] |  | 30.89 | | 7.33* | 0.24 | [6.85, 7.81] |  | | 20.01 | | 5.86* | | 0.29 | | [5.27, 6.46] |
| [50:50] | 12.23 | | 3.65* | | 0.30 | | [3.05, 4.26] | |  | 14.00 | 4.09* | 0.29 | [3.50, 4.68] |  | 14.33 | | 4.94* | 0.35 | [4.25, 5.64] |  | | 15.46 | | 4.09* | | 0.26 | | [3.55, 4.62] |
| [70:30] | 8.52 | | 1.89* | | 0.22 | | [1.44, 2.34] | |  | 15.84 | 3.08* | 0.19 | [2.68, 3.47] |  | 15.83 | | 3.15* | 0.20 | [2.75, 3.55] |  | | 15.39 | | 3.75* | | 0.24 | | [3.25, 4.24] |
| **Fea-hap** | |  | |  | |  | |  |  |  |  |  |  |  |  |  | |  |  | |  |  |  | |  | |  | |
| [30:70] | | 21.14 | | 6.30* | | 0.30 | | [5.70, 6.91] |  | 25.87 | 7.14* | 0.28 | [6.58, 7.70] |  | 28.74 | 7.70* | | 0.27 | [7.16, 8.24] | |  | 19.07 | 6.23* | | 0.33 | | [5.57, 6.89] | |
| [50:50] | | 12.96 | | 3.81* | | 0.29 | | [3.22, 4.41] |  | 15.54 | 4.25* | 0.27 | [3.69, 4.80] |  | 15.99 | 5.10* | | 0.32 | [4.46, 5.75] | |  | 16.84 | 4.24* | | 0.25 | | [3.73, 4.75] | |
| [70:30] | | 5.78 | | 1.30* | | 0.23 | | [0.85, 1.76] |  | 15.44 | 2.49* | 0.16 | [2.16, 2.81] |  | 12.11 | 2.56* | | 0.21 | [2.13, 2.99] | |  | 11.59 | 3.16* | | 0.27 | | [2.61, 3.71] | |

*Note. N = 39*. Abbreviations: ang = anger, disg = disgust, fea = fear, hap = happiness, sad = sadness. 30:70 = The first emotion is intended to be less prominent than the second emotion. 50:50 = Both emotions are intended to be equally prominent. 70:30 = The first emotion is intended to be more prominent than the second emotion. Asterisks indicate significance after correction (Bonferroni corrected alpha level = .0021): * *p* < .001.

## Table S4

*Multiple Pairwise Comparisons for 30:70 and 70:30 Proportions Between Rating Scales for Intended Emotion Combinations (Study 2)*

| Emotion Scales  [Emotion Proportion] | *t*(38) | *M* ^diff^ | *SE* | CI 95% |
| --- | --- | --- | --- | --- |
| Anger vs Disgust |  |  |  |  |
| Ang-disg [30:70] | -11.75 | -3.62** | 0.31 | [-4.25, -3.00] |
| Ang-disg [70:30] | 9.90 | 2.74** | 0.25 | [2.18, 3.30] |
| Anger vs Fear |  |  |  |  |
| Ang-fea [30:70] | 3.59 | 1.11* | 0.31 | [0.48, 1.73] |
| Ang-fea [70:30] | 17.52 | 5.82** | 0.28 | [5.15, 6.49] |
| Anger vs Happiness |  |  |  |  |
| Ang-hap [30:70] | -13.32 | -4.62** | 0.35 | [-5.32, -3.92] |
| Ang-hap [70:30] | 9.53 | 2.91** | 0.34 | [2.29, 3.52] |
| Anger vs Sadness |  |  |  |  |
| Ang-sad [30:70] | -6.07 | -1.53** | 0.25 | [-2.05, -1.02] |
| Ang-sad [70:30] | 15.49 | 4.06** | 0.31 | [3.53, 4.59] |
| Disgust vs Fear |  |  |  |  |
| Disg-fea [30:70] | -7.63 | -2.13** | 0.28 | [-2.70, -1.57] |
| Disg-fea [70:30] | 18.87 | 6.42** | 0.35 | [5.73, 7.11] |
| Disgust vs Happiness |  |  |  |  |
| Disg-hap [30:70] | -15.28 | -5.15** | 0.34 | [-5.83, -4.47] |
| Disg-hap [70:30] | 8.30 | 3.52** | 0.25 | [2.66, 4.38] |
| Disgust vs Sadness |  |  |  |  |
| Disg-sad [30:70] | -21.93 | -5.99** | 0.31 | [-6.54, -5.44] |
| Disg-sad [70:30] | 0.54 | 0.14 | 0.28 | [-0.38, 0.65] |
|  |  |  |  |  |
|  |  |  |  |  |
| **Table S4 (Continued)** |  |  |  |  |
| Emotion Scales  [Emotion Proportion] | *t*(38) | *M* ^diff^ | *SE* | CI 95% |
| Fear vs Happiness |  |  |  |  |
| Fea-hap [30:70] | -13.55 | -4.76** | 0.31 | [-5.47, -4.05] |
| Fea-hap [70:30] | 7.18 | 2.32** | 0.34 | [1.67, 2.98] |
| Fear vs Sadness |  |  |  |  |
| Fea-sad [30:70] | -18.74 | -5.55** | 0.31 | [-6.15, -4.95] |
| Fea-sad [70:30] | 6.11 | 1.82** | 0.31 | [1.21, 2.42] |
| Happiness vs Sadness |  |  |  |  |
| Hap-sad [30:70] | -14.51 | -4.39** | 0.35 | [-5.01, -3.78] |
| Hap-sad [70:30] | 3.21 | 1.15 | 0.35 | [0.43, 1.88] |

*Note.* *N* = 39. Abbreviations: ang = anger, disg = disgust, fea = fear, hap = happiness, sad = sadness. 30:70 = The first emotion is intended to be less prominent than the second emotion. 50:50 = Both emotions are intended to be equally prominent. 70:30 = The first emotion is intended to be more prominent than the second emotion. Asterisks indicate significance after correction (Bonferroni corrected alpha level = .0025): * *p* < .0025, ** *p* < .00001.

## Table S5

*Scale Usage across all Rating Scales for each Emotion Combination and Proportion Condition (Study 2).*

|  |  |  |  |  |  |  | Usage of Scales in Proportions (M/SD) | | | | | | | | | | | | | | | | | | | | |  |  |  |  |  | Number of used Scales |
| --- | --- | --- | --- | --- | --- | --- | --- | --- | --- | --- | --- | --- | --- | --- | --- | --- | --- | --- | --- | --- | --- | --- | --- | --- | --- | --- | --- | --- | --- | --- | --- | --- | --- |
| Emotion Combination |  |  |  |  |  |  | One Scale |  |  |  |  | Two Scales |  |  |  |  | Three Scales |  |  |  |  | Four Scales |  |  |  |  | Five Scales |  |  |  |  |  | (M/SD) |
| Ang-disg |  |  |  |  |  |  |  |  |  |  |  |  |  |  |  |  |  |  |  |  |  |  |  |  |  |  |  |  |  |  |  |  |  |
| 30:70 |  |  |  |  |  |  | 0.303 (0.462) |  |  |  |  | **0.551 (0.500)** |  |  |  |  | 0.124 (0.331) |  |  |  |  | 0.021 (0.145) |  |  |  |  | 0.000 (0.000) |  |  |  |  |  | 1.86 (0.60) |
| 50:50 |  |  |  |  |  |  | 0.256 (0.439) |  |  |  |  | **0.581 (0.496)** |  |  |  |  | 0.141 (0.350) |  |  |  |  | 0.021 (0.145) |  |  |  |  | 0.000 (0.000) |  |  |  |  |  | 1.93 (0.60) |
| 70:30 |  |  |  |  |  |  | 0.346 (0.478) |  |  |  |  | **0.521 (0.502)** |  |  |  |  | 0.111 (0.316) |  |  |  |  | 0.021 (0.145) |  |  |  |  | 0.000 (0.000) |  |  |  |  |  | 1.81 (0.66) |
| Ang-fea |  |  |  |  |  |  |  |  |  |  |  |  |  |  |  |  |  |  |  |  |  |  |  |  |  |  |  |  |  |  |  |  |  |
| 30:70 |  |  |  |  |  |  | 0.261 (0.441) |  |  |  |  | **0.517 (0.502)** |  |  |  |  | 0.188 (0.393) |  |  |  |  | 0.026 (0.159) |  |  |  |  | 0.009 (0.093) |  |  |  |  |  | 2.00 (0.72) |
| 50:50 |  |  |  |  |  |  | 0.359 (0.482) |  |  |  |  | **0.517 (0.502)** |  |  |  |  | 0.098 (0.299) |  |  |  |  | 0.026 (0.159) |  |  |  |  | 0.000 (0.000) |  |  |  |  |  | 1.79 (0.62) |
| 70:30 |  |  |  |  |  |  | **0.483 (0.502)** |  |  |  |  | 0.380 (0.488) |  |  |  |  | 0.124 (0.331) |  |  |  |  | 0.013 (0.113) |  |  |  |  | 0.000 (0.000) |  |  |  |  |  | 1.67 (0.69) |
| Ang-hap |  |  |  |  |  |  |  |  |  |  |  |  |  |  |  |  |  |  |  |  |  |  |  |  |  |  |  |  |  |  |  |  |  |
| 30:70 |  |  |  |  |  |  | 0.256 (0.439) |  |  |  |  | **0.615 (0.489)** |  |  |  |  | 0.124 (0.331) |  |  |  |  | 0.004 (0.066) |  |  |  |  | 0.000 (0.000) |  |  |  |  |  | 1.88 (0.59) |
| 50:50 |  |  |  |  |  |  | 0.141 (0.350) |  |  |  |  | **0.722 (0.450)** |  |  |  |  | 0.132 (0.341) |  |  |  |  | 0.004 (0.066) |  |  |  |  | 0.000 (0.000) |  |  |  |  |  | 2.00 (0.51) |
| 70:30 |  |  |  |  |  |  | 0.235 (0.426) |  |  |  |  | **0.577 (0.497)** |  |  |  |  | 0.154 (0.363) |  |  |  |  | 0.030 (0.171) |  |  |  |  | 0.004 (0.066) |  |  |  |  |  | 1.99 (0.64) |
| Ang-sad |  |  |  |  |  |  |  |  |  |  |  |  |  |  |  |  |  |  |  |  |  |  |  |  |  |  |  |  |  |  |  |  |  |
| 30:70 |  |  |  |  |  |  | 0.115 (0.321) |  |  |  |  | **0.650 (0.480)** |  |  |  |  | 0.188 (0.393) |  |  |  |  | 0.038 (0.193) |  |  |  |  | 0.009 (0.093) |  |  |  |  |  | 2.18 (0.63) |
| 50:50 |  |  |  |  |  |  | 0.107 (0.311) |  |  |  |  | **0.590 (0.495)** |  |  |  |  | 0.265 (0.444) |  |  |  |  | 0.038 (0.193) |  |  |  |  | 0.000 (0.000) |  |  |  |  |  | 2.24 (0.59) |
| 70:30 |  |  |  |  |  |  | 0.321 (0.469) |  |  |  |  | **0.457 (0.501)** |  |  |  |  | 0.205 (0.406) |  |  |  |  | 0.017 (0.130) |  |  |  |  | 0.000 (0.000) |  |  |  |  |  | 1.92 (0.69) |
| Disg-fea |  |  |  |  |  |  |  |  |  |  |  |  |  |  |  |  |  |  |  |  |  |  |  |  |  |  |  |  |  |  |  |  |  |
| 30:70 |  |  |  |  |  |  | 0.248 (0.434) |  |  |  |  | **0.551 (0.500)** |  |  |  |  | 0.184 (0.389) |  |  |  |  | 0.017 (0.130) |  |  |  |  | 0.000 (0.000) |  |  |  |  |  | 1.97 (0.66) |
| 50:50 |  |  |  |  |  |  | 0.162 (0.371) |  |  |  |  | **0.607 (0.491)** |  |  |  |  | 0.209 (0.409) |  |  |  |  | 0.017 (0.130) |  |  |  |  | 0.004 (0.066) |  |  |  |  |  | 2.09 (0.61) |
| 70:30 |  |  |  |  |  |  | 0.342 (0.477) |  |  |  |  | **0.504 (0.503)** |  |  |  |  | 0.141 (0.350) |  |  |  |  | 0.013 (0.113) |  |  |  |  | 0.000 (0.000) |  |  |  |  |  | 1.82 (0.66) |
|  |  |  |  |  |  |  |  |  |  |  |  |  |  |  |  |  |  |  |  |  |  |  |  |  |  |  |  |  |  |  |  |  |  |
| **Table S5 (Continued)** |  |  |  |  |  |  |  | | | | | | | | | | | | | | | | | | | | |  |  |  |  |  |  |
|  |  |  |  |  |  |  | Usage of Scales in Proportions (M/SD) | | | | | | | | | | | | | | | | | | | | |  |  |  |  |  | Number of used Scales |
| Emotion Combination |  |  |  |  |  |  | One Scale |  |  |  |  | Two Scales |  |  |  |  | Three Scales |  |  |  |  | Four Scales |  |  |  |  | Five Scales |  |  |  |  |  | (M/SD) |
| Disg-hap |  |  |  |  |  |  |  |  |  |  |  |  |  |  |  |  |  |  |  |  |  |  |  |  |  |  |  |  |  |  |  |  |  |
| 30:70 |  |  |  |  |  |  | 0.167 (0.375) |  |  |  |  | **0.667 (0.474)** |  |  |  |  | 0.158 (0.367) |  |  |  |  | 0.004 (0.066) |  |  |  |  | 0.004 (0.066) |  |  |  |  |  | 2.01 (0.59) |
| 50:50 |  |  |  |  |  |  | 0.103 (0.305) |  |  |  |  | **0.705 (0.458)** |  |  |  |  | 0.171 (0.379) |  |  |  |  | 0.021 (0.145) |  |  |  |  | 0.000 (0.000) |  |  |  |  |  | 2.11 (0.55) |
| 70:30 |  |  |  |  |  |  | 0.167 (0.375) |  |  |  |  | **0.526 (0.502)** |  |  |  |  | 0.278 (0.450) |  |  |  |  | 0.026 (0.159) |  |  |  |  | 0.004 (0.066) |  |  |  |  |  | 2.18 (0.66) |
| Disg-sad |  |  |  |  |  |  |  |  |  |  |  |  |  |  |  |  |  |  |  |  |  |  |  |  |  |  |  |  |  |  |  |  |  |
| 30:70 |  |  |  |  |  |  | 0.380 (0.488) |  |  |  |  | **0.440 (0.499)** |  |  |  |  | 0.154 (0.363) |  |  |  |  | 0.026 (0.159) |  |  |  |  | 0.000 (0.000) |  |  |  |  |  | 1.82 (0.71) |
| 50:50 |  |  |  |  |  |  | 0.141 (0.350) |  |  |  |  | **0.530 (0.502)** |  |  |  |  | 0.303 (0.462) |  |  |  |  | 0.026 (0.159) |  |  |  |  | 0.000 (0.000) |  |  |  |  |  | 2.21 (0.63) |
| 70:30 |  |  |  |  |  |  | 0.308 (0.464) |  |  |  |  | **0.479 (0.502)** |  |  |  |  | 0.175 (0.382) |  |  |  |  | 0.038 (0.193) |  |  |  |  | 0.000 (0.000) |  |  |  |  |  | 1.94 (0.76) |
| Fea-hap |  |  |  |  |  |  |  |  |  |  |  |  |  |  |  |  |  |  |  |  |  |  |  |  |  |  |  |  |  |  |  |  |  |
| 30:70 |  |  |  |  |  |  | 0.261 (0.441) |  |  |  |  | **0.650 (0.480)** |  |  |  |  | 0.085 (0.281) |  |  |  |  | 0.004 (0.066) |  |  |  |  | 0.000 (0.000) |  |  |  |  |  | 1.83 (0.58) |
| 50:50 |  |  |  |  |  |  | 0.158 (0.367) |  |  |  |  | **0.671 (0.472)** |  |  |  |  | 0.167 (0.375) |  |  |  |  | 0.004 (0.066) |  |  |  |  | 0.000 (0.000) |  |  |  |  |  | 2.02 (0.57) |
| 70:30 |  |  |  |  |  |  | 0.192 (0.396) |  |  |  |  | **0.590 (0.495)** |  |  |  |  | 0.188 (0.393) |  |  |  |  | 0.026 (0.159) |  |  |  |  | 0.004 (0.066) |  |  |  |  |  | 2.06 (0.66) |
| Fea-sad |  |  |  |  |  |  |  |  |  |  |  |  |  |  |  |  |  |  |  |  |  |  |  |  |  |  |  |  |  |  |  |  |  |
| 30:70 |  |  |  |  |  |  | 0.457 (0.501) |  |  |  |  | **0.466 (0.502)** |  |  |  |  | 0.073 (0.261) |  |  |  |  | 0.004 (0.066) |  |  |  |  | 0.000 (0.000) |  |  |  |  |  | 1.62 (0.61) |
| 50:50 |  |  |  |  |  |  | 0.252 (0.437) |  |  |  |  | **0.581 (0.496)** |  |  |  |  | 0.137 (0.345) |  |  |  |  | 0.030 (0.171) |  |  |  |  | 0.000 (0.000) |  |  |  |  |  | 1.94 (0.61) |
| 70:30 |  |  |  |  |  |  | 0.329 (0.472) |  |  |  |  | **0.521 (0.502)** |  |  |  |  | 0.124 (0.331) |  |  |  |  | 0.017 (0.130) |  |  |  |  | 0.009 (0.093) |  |  |  |  |  | 1.85 (0.73) |
| Hap-sad |  |  |  |  |  |  |  |  |  |  |  |  |  |  |  |  |  |  |  |  |  |  |  |  |  |  |  |  |  |  |  |  |  |
| 30:70 |  |  |  |  |  |  | 0.363 (0.484) |  |  |  |  | **0.496 (0.503)** |  |  |  |  | 0.115 (0.321) |  |  |  |  | 0.026 (0.159) |  |  |  |  | 0.000 (0.000) |  |  |  |  |  | 1.80 (0.69) |
| 50:50 |  |  |  |  |  |  | 0.192 (0.396) |  |  |  |  | **0.705 (0.458)** |  |  |  |  | 0.085 (0.281) |  |  |  |  | 0.017 (0.130) |  |  |  |  | 0.000 (0.000) |  |  |  |  |  | 1.93 (0.53) |
| 70:30 |  |  |  |  |  |  | 0.171 (0.379) |  |  |  |  | **0.726 (0.448)** |  |  |  |  | 0.094 (0.293) |  |  |  |  | 0.009 (0.093) |  |  |  |  | 0.000 (0.000) |  |  |  |  |  | 1.94 (0.48) |

*N* = 39. Abbreviations: ang = anger, disg = disgust, fea = fear, hap = happiness, sad = sadness. 30:70 = The first emotion is intended to be less prominent than the second emotion. 50:50 = Both emotions are intended to be equally prominent. 70:30 = The first emotion is intended to be more prominent than the second emotion. Values in bold indicate the greatest proportion of scale usage.
